# High individual repeatability of the migratory behaviour of a long-distance migratory seabird

**DOI:** 10.1186/s40462-022-00303-y

**Published:** 2022-02-05

**Authors:** Nathalie Kürten, Heiko Schmaljohann, Coraline Bichet, Birgen Haest, Oscar Vedder, Jacob González-Solís, Sandra Bouwhuis

**Affiliations:** 1 Institute of Avian Research, An der Vogelwarte 21, 26386 Wilhelmshaven, Germany; 2 Institute of Biology and Environmental Sciences, University of Oldenburg, Carl-von-Ossietzky-Str. 9–11, 26129 Oldenburg, Germany; 3 Centre d’Etudes Biologiques de Chizé, UMR 7372, CNRS-Université de La Rochelle, 79360 Villiers-en-Bois, France; 4 Department of Bird Migration, Swiss Ornithological Institute, 6204 Sempach, Switzerland; 5 Institut de Recerca de la Biodiversitat and Departament de Biologia Evolutiva, Ecologia i Ciències Ambientals, Universitat de Barcelona, Av. Diagonal 643, 08028 Barcelona, Spain

**Keywords:** Animal movement, Bird migration, Migratory behaviour, Individual consistency, Repeatability, Geolocation, Spatial ecology, Phenology

## Abstract

**Background:**

Understanding the evolution of migration requires knowledge of the patterns, sources, and consequences of variation in migratory behaviour, a need exacerbated by the fact that many migratory species show rapid population declines and require knowledge-based conservation measures. We therefore need detailed knowledge on the spatial and temporal distribution of individuals across their annual cycle, and quantify how the spatial and temporal components of migratory behaviour vary within and among individuals.

**Methods:**

We tracked 138 migratory journeys undertaken by 64 adult common terns (*Sterna hirundo*) from a breeding colony in northwest Germany to identify the annual spatiotemporal distribution of these birds and to evaluate the individual repeatability of eleven traits describing their migratory behaviour.

**Results:**

Birds left the breeding colony early September, then moved south along the East Atlantic Flyway. Wintering areas were reached mid-September and located at the west and south coasts of West Africa as well as the coasts of Namibia and South Africa. Birds left their wintering areas late March and reached the breeding colony mid-April. The timing, total duration and total distance of migration, as well as the location of individual wintering areas, were moderately to highly repeatable within individuals (repeatability indexes: 0.36–0.75, 0.65–0.66, 0.93–0.94, and 0.98–1.00, respectively), and repeatability estimates were not strongly affected by population-level inter-annual variation in migratory behaviour.

**Conclusions:**

We found large between-individual variation in common tern annual spatiotemporal distribution and strong individual repeatability of several aspects of their migratory behaviour.

**Supplementary Information:**

The online version contains supplementary material available at 10.1186/s40462-022-00303-y.

## Background

Migration constitutes an important part of the annual cycle of many species across taxa [1, 2]. Spatiotemporal patterns, however, vary remarkably among species and populations, as well as among and within individuals within populations [1]. Such variation should generally reflect differences in the selective ecological conditions experienced by these species, populations, or individuals [3], but detailed knowledge of the patterns, sources, and consequences of variation in migratory behaviour is needed to understand the evolution of migration [4, 5]. This need is exacerbated by the fact that many migratory species show rapid population declines (e.g. [6]), such that we are in urgent need of knowledge-based conservation measures to protect decreasing populations (e.g. [7, 8]).

To understand the causes and consequences of variation in migratory behaviour, we need detailed information on the spatial and temporal distribution of individuals across their annual cycle. Ideally, this information should comprise a description of both the between-individual variation in, and within-individual consistency of, migratory behaviour. This knowledge can then be used to (i) learn whether migratory behaviour is underpinned by (epi)genetic heritability, developmental plasticity or phenotypic flexibility [4, 5, 9], (ii) assess the consequences of migratory behaviour at different timescales by quantifying seasonal, multi-seasonal or multi-year carry-over effects [10, 11] on fitness parameters (e.g. [12]), and (iii) inform or improve conservation management (see [13, 14]).

Between-individual variation in behaviour is widespread in natural populations [15, 16]. With respect to migratory behaviour, most studies pertain to birds. Studies on raptors (e.g. [17]), songbirds (e.g. [18]), geese (e.g. [19]), shorebirds (e.g. [20]), and seabirds (e.g. [21]) have reported varying levels of between-individual variation in migratory behaviour, linking it to traits such as age (e.g. [22, 23]) or sex (e.g. [24–26]). Juvenile honey buzzards (*Pernis apivorus*), for example, were found to migrate slower than adults [22] and female black-browed albatrosses (*Thalassarche melanophris*) were found to winter further north than males [25].

Variation in migratory behaviour can also occur within individuals [1]. The within-individual consistency of behaviour is often calculated using the repeatability index “*R*” [27], which is defined as the proportion of the total variation in a trait that can be attributed to differences between (compared to within) individuals [28]. As for the between-individual variation, the repeatability of migratory behaviour has been quantified across taxa (e.g. reptiles [29]), fish [30], and insects [31]), albeit most frequently in birds (reviewed by [32, 33]). Here, studies on raptors (e.g. [34, 35]), shorebirds (e.g. [36–38]), geese (e.g. [19, 39]), songbirds (e.g. [40, 41]) and seabirds (e.g. [42–44]) have reported repeatabilities as low as 0.03 for the duration of migration in Scopoli`s shearwater (*Calonectris diomedea*) [42], or as high as 0.99 for stopover sites (longitude and latitude) in oriental honey buzzards [35]. The observed variability in repeatabilities between traits, seasons, populations and species shows that general conclusions are hard to draw and that any study aiming at understanding the extent, causes, and consequences of variation in migratory behaviour needs to first quantify variation within and between individuals.

Here, we report on a 5-year study in which we tracked 138 migratory journeys undertaken by 64 common terns (*Sterna hirundo*) from a breeding colony in northwest Germany. Although internationally the common tern is listed as being of least concern [45], in Germany it is locally endangered [46], and understanding its migratory behaviour may help to learn why. Using one to four tracks per individual, we therefore (i) identify the general spatiotemporal distribution during the annual cycle, (ii) evaluate the individual repeatability of eleven traits describing their migratory behaviour, and (iii) assess whether our repeatability estimates are affected by correcting for annual variation in migratory behaviour at the population level.

## Methods

### Study species and site

Common terns are Holarctic colonially breeding and long-distance migratory seabirds [47]. They display high breeding site fidelity (adult local return rate is ca. 90%; [48, 49]), are relatively easily caught during incubation, and are large enough to carry small tracking devices without detectable detrimental effects on their reproductive performance or survival [50]. This facilitates efficient use of such devices to monitor the (repeated) migratory behaviour of many individuals.

We studied the migratory behaviour of common terns breeding at a monospecific colony located at the Banter See in Wilhelmshaven, at the German North Sea coast (53° 30′ 40″ N, 08° 06′ 20″ E). This colony is the focus of a long-term individual-based study, in which all local fledglings have been marked with a transponder since 1992, allowing for automatic and remote detection of recruits using an antenna system (for more details see [51]). The sex of tracked birds has been molecularly determined following Becker & Wink [52].

### Deployment and recovery of light-level geolocators

Between mid-May and early July 2016–2019, we used the antenna system to identify 24, 36, 50, and 54 focal common terns of both sexes as they incubated their clutches (see Additional file 1: Table S1). We caught them using an electronically released drop trap, on average 17 days ± 3.4 SD after the first egg was laid. Before initial capture (and for birds carrying a tracking device from a previous year, on the day of laying), eggs were replaced by model eggs to prevent potential catching- (or tag-) induced damage to the real eggs [53]. Real eggs were incubated using digital incubators (Rcom max 50 and Rcom pro 20; Autoelex Co., Ltd., South Korea; [54]). The captured birds were weighed (average body mass: 129.2 g ± 7.8 SD) using a digital balance (± 1.0 g accuracy; MAULalpha, Jakob Maul GmbH, Germany) and tagged with a light-level geolocator (Intigeo-C65, Migrate Technology, UK). The geolocator was attached to the leg of the bird using a 10 mm aluminium ring. The total mass of the ring, glue and geolocator was 1.6 g, i.e. 1.2% ± 0.1 SD of the body mass of the birds at tagging and below the recommended threshold of 3% [55]. Tags did not have a detectable effect on the behaviour, reproductive performance, or survival of the birds [50]. Total handling time was 5.8 min ± 2.7 SD and all birds resumed normal incubation after being handled (i.e. no clutch was abandoned).

In the breeding seasons of 2017–2020, we recaptured 22, 29, 42, and 49 of the ‘geolocator birds’ that returned to remove the geolocator and extract the data. The other birds either did not survive or return to the colony (n = 16), returned but did not attempt to reproduce, i.e. were impossible to catch (n = 4), or had lost their geolocator (n = 2). Out of the 142 geolocators that we retrieved from 30 males and 35 females, 105 (74%) were still recording at recapture, 2 (1%) stopped working during spring migration, 31 (22%) stopped working in the wintering area and 4 (3%) did not record any data (see Additional file 1: Table S1 for more details).

### Analysis of light-level geolocation data

We set our geolocators to sample ambient light intensity every minute and to archive the maximum light intensity every 5 min (mode 10, Migrate Technology). The retrieved light intensity data were analysed using the software R (version 4.0.3, [56]) and the R package “*FLightR*” (version 0.5.0, [57]), following the workflow detailed in the supplementary material from Rakhimberdiev et al. [58].

First, daily sunrise and sunset (i.e. twilight events) were identified with the function “*preprocessLight*” of the R package “*BAStag*” [59] using a light-level threshold of 1.5. Extreme outliers (e.g. > 30 min difference with the previous and subsequent twilight) were adjusted or excluded by means of a visual inspection of each individual sunrise and sunset. Next, calibration periods were individually determined by visual inspection of the output plotted using the “*plot_slopes_by_location*” function in FLightR [60], using the weeks the birds were known to be at the breeding colony (i.e. “on-bird” calibration). The first calibration period started after incubation (a period of high shading) and ended prior to migration. For geolocators that still recorded data at recapture, we defined a second calibration period, which started after the return to the colony and ended at the onset of incubation.

For the subsequent movement analyses, location estimates were not restricted to either land or sea (as terns may migrate over-sea and across-land [61]), but positions were spatially constrained using the function “*make.grid*” to the area between 40° W, 45° S, 45° E and 65° N to adhere to ring recovery data [53]. We also limited the maximum flight distance between twilights to 1200 ± 300 km, i.e. 24 h times 50 km/h [62]. Finally, the “*run.particle.filter*” function was set to 1 million particles to optimize the track of each individual and its uncertainty [60] (the R code of the analysis is provided in the supplementary information – see Additional file 2).

### Defining migration traits

We defined each individual’s year-specific migratory and stationary periods (stopover site(s) and wintering area(s)) using the “*stationary.migration.summary*” function of “*FLightR*”. To detect migratory periods, we set the minimum probability that defines movement (“*prob.cutoff*”) to 0.4 (see Additional file 1: Tables S2 and S3). Stationary periods are assigned by the function if an individual stays in a given area for a minimum number of days, set using the argument “*min.stay*”. For the terns, we set “*min.stay*” to ten twilights, i.e. five days, as common terns moving along the East Atlantic Flyway are thought to use stopover sites for more than five consecutive days [53, 63]. Detected stopover sites within a range of 250 km around the breeding colony were discarded (n = 10 stopover sites of 9 individuals) to account for potential movements within the breeding period, including local movement to nearby locations used after breeding and loafing (i.e. after breeding failure, but before migration).

From the output provided by the “*stationary.migration.summary*” function, we extracted the longitude and latitude of each stopover site and each wintering area. Due to an uncertainty of dates of arrival to, and departure from, the stopover site(s) (i.e. no length of stay at stopover site(s) calculable; see Additional file 1: Tables S2 and S3), we only extracted the dates of arrival to, and departure from, the breeding colony and each wintering area. The length of stay at the wintering area(s) was calculated by subtracting the arrival date at the wintering area(s) from the departure date from the wintering area(s). Wintering area(s) were defined as sites south of 27° N (the Canary Islands, located at 28° N, are a well-known stopover area for common terns [53]) at which individuals stayed more than two months. Since birds are not totally stationary within their wintering area(s), the function detected movements in 19 tracks from 16 individuals and split their wintering area(s) into two or more small wintering areas, characterised by a small distance between them (average: 252 km; with a maximum cut-off of 500 km) and overlapping departure and arrival dates. In these cases, we manually merged the wintering areas and calculated the location of the resulting overall wintering area by using all available positions between arrival at the first, and departure from the last, wintering area. In an additional 5 tracks from 4 individuals, however, the distance between wintering areas was > 500 km and the departure and arrival dates did not overlap, such that we retained both wintering areas (see Results).

The total migration distance (km) covered by an individual in a given year was calculated as the great circle distance (i.e. the shortest distance between two points; orthodrome) between the breeding area, any potential stopover site(s) and the wintering area(s) or vice versa using the function “*distVincentyEllipsoid*” of the package “*geosphere*” [64]. We used the great circle distance instead of the rhumb line (loxodrome), because (i) the inherent error associated with the measurement is the same for every bird, and (ii) there was only a marginal difference between these two calculations (0.47% and 0.40% for total migration distance in autumn and spring, respectively). For the 5 cases in which 4 individuals had more than one wintering area, the total migration distance was calculated to the first wintering area for autumn migration and from the last wintering area for spring migration.

We considered two different estimates of migration duration and speed. The total duration of migration (days) was estimated by subtracting the arrival date from the departure date during autumn and spring migration. The actual duration of migration (days) was estimated similarly, but using only tracks of birds for which no stopover sites were detected. The total speed of migration (km/day) was calculated by dividing the total migration distance by the total duration of migration. The (minimum) travel speed (km/day) was calculated by dividing the total migration distance by the actual duration of migration.

Migration schedules were summarized using mean values of the longitude and latitude for location estimates, mean values for estimates of the length of stay at the wintering area(s) (days), the dates of departure and arrival, total migration distance (km), total duration of migration (days), actual duration of migration (days), total speed of migration (km/day) and travel speed (km/day), and the range for the number of stopover sites.

### Statistical analyses

For autumn migration, our dataset comprised 138 tracks from 64 birds. For the wintering area, where 31 geolocators stopped working (see above), we could still analyse the longitude and latitude using all 138 tracks, as our estimates of the repeatability of longitude and latitude did not differ when we excluded tracks based on data for less than two months (see Additional file 1: Table S4). For the length of stay and departure date from the wintering area, however, our dataset comprised 107 tracks from 60 birds. For the remaining traits during spring migration, our dataset comprised 105 tracks from 60 birds.

#### Spatiotemporal distribution

We first tested whether migratory traits differed between autumn and spring migration and/or between the sexes by running a set of (generalised) linear mixed models that included season and sex as two 2-level categorical fixed effects. We also included the interaction between sex and season, but removed it if not significant to allow for a more straightforward interpretation of the main effects. All models included year as a categorical fixed effect and individual identity as a random intercept to account for between-year variation and the non-independence of repeated tracks from the same birds, respectively. Variation in total migration distance, total duration of migration, actual duration of migration, total speed of migration and travel speed were analysed using five linear mixed models, since log transformation of the data facilitated the fit of a normal error distribution. Variation in stopover probability and the number of stopover sites were analysed using two generalized linear mixed models with a binominal error distribution and a “*logit*” link function, and a Poisson distribution and a "*log*" link function, respectively.

Second, we tested for sex differences in the departure date from the colony, arrival date at the (first) wintering area, departure date from the (last) wintering area, arrival date at the colony, longitude and latitude of the (first) wintering area (all not season-dependent) using six linear mixed models with a normal error distribution. These models also included year as a categorical fixed effect and individual identity as a random intercept.

The models were run with the functions “*lmer*” and “*glmer*” of the R package “*lme4*” [65]. P-values for linear mixed models were obtained using the “*lmerTest*” package [66], those for generalised linear mixed models were extracted from the “*glmer*” summary output. The level of significance was set to *p* < 0.05 and parameter estimates are given as mean ± SE.

#### Repeatability of the spatiotemporal distribution

The intra-individual repeatability “*R*” was calculated for spring and autumn total migration distance, spring and autumn total duration of migration, autumn departure date from the breeding colony, autumn arrival date at the (first) wintering area, longitude of the (first) wintering area, latitude of the (first) wintering area, length of stay at the (first) wintering area, spring departure date from the (last) wintering area and spring arrival date at the breeding colony using eleven models, each fitted with a normal error distribution, year and sex as categorical fixed effects and individual identity as a random intercept. In addition, we calculated the repeatability without accounting for year as a fixed effect. While analyses “*with year*” provide information on the repeatability of the relative trait expression of an individual in comparison to its conspecifics (e.g. whether an individual generally departs earlier than its conspecifics), analyses “*without year*” provide information on the repeatability of its absolute trait expression (e.g. whether an individual generally departs on a similar day of the year, while its conspecifics depart on another day of the year).

We also tried to calculate the intra-individual repeatability of stopover probability during autumn and spring migration using models fitted with a binominal error distribution and “*logit*” link function. These models again included (year and) sex as (a) categorical fixed effect(s) and individual identity as a random intercept. Since these models, however, did not produce reliable output (see Additional file 1: Table S5), we instead investigated the output provided by the “*stationary.migration.summary*” function of individuals for which we had full data for two or more years, and report the number of individuals that showed consistent or inconsistent stopover behaviour.

**Fig. 1:**
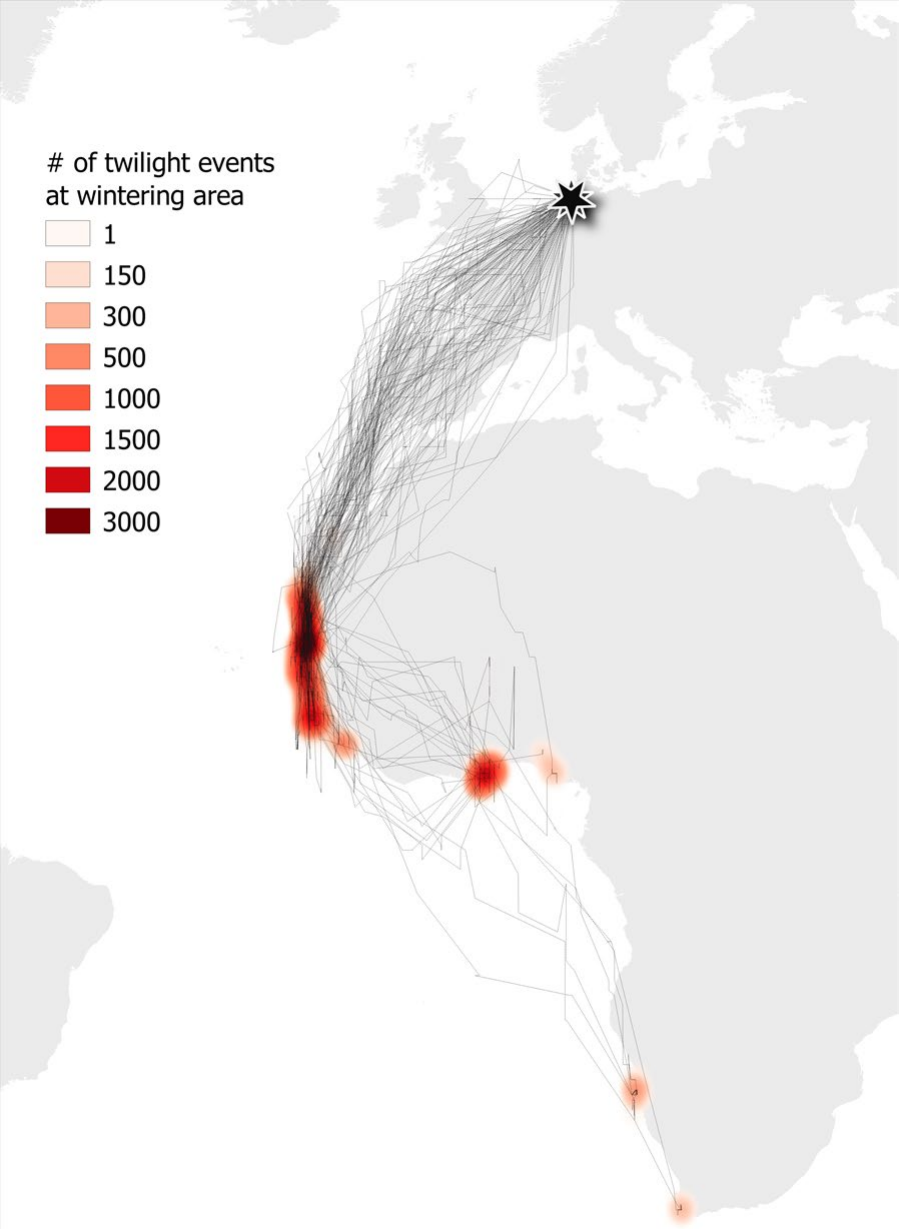
Migration routes and wintering areas of 64 common terns tracked with a light-level geolocator. Wintering areas are based on the individuals’ locations (estimated using FLightR) at daily twilights from one week after the estimated arrival up to one week before the estimated departure from the wintering area. For the birds of which the geolocator stopped working at the wintering area, data were used until the last estimated position. The heatmaps of the twilight positions were produced in QGIS using quartic kernel density with a 2º radius

All repeatability models were run using the “*rpt*” and the “*rptBinary*” functions of the R package “r*ptR”* [67], which provided mean (± SE) R estimates, 95% confidence intervals obtained from 1000 bootstrap iterations and p-values. We visualised our data using the R package “*ggplot2”* [68] and the program QGIS [69] making use of the “*heatmap*” function (quartic kernel with a radius of 2 degrees, and an output grid size of 0.1 degrees; Figs. 1, 3).

## Results

### Spatiotemporal distribution

With respect to the *average* spatiotemporal distribution, common terns left the breeding colony in the northwest of Germany on 5 September (range 24 July–1 October), with females leaving earlier than males (Table 1, pt. A). Birds of both sexes moved south along the East Atlantic Flyway, flying both over-sea and across-land (Fig. 1). In 49 tracks of 26 individuals, 1–2 autumn stopover sites at the coasts of France, Portugal, Morocco, Western Sahara or Mauretania, or at the Canary Islands were used (see Additional file 1: Table S6).

**Table 1:** Summary of models testing for effects of sex (female as a reference) on (A) departure from colony, (B) arrival at wintering area, (C) wintering longitude, (D) wintering latitude, (E) wintering latitude and (F) arrival at colony of common terns deployed with geolocators during 2016–2020

Model	estimate ± SE	*df*	t-value	p-value
**A. departure from colony (days)**^**a**^				
Intercept	233.73 ± 2.70	120.49	86.64	**< 0.001**
year_2017/18	4.16 ± 2.15	76.01	1.93	0.057
year_2018/19	12.82 ± 2.10	79.59	6.11	**< 0.001**
year_2019/20	11.36 ± 2.17	86.55	5.24	**< 0.001**
sex	13.19 ± 3.09	73.58	4.27	**< 0.001**
**B. arrival at wintering area (days)**^**a**^				
Intercept	243.86 ± 3.64	125.43	67.09	**< 0.001**
year_2017/18	7.13 ± 3.18	79.23	2.25	**0.028**
year_2018/19	12.60 ± 3.08	83.93	4.09	**< 0.001**
year_2019/20	14.75 ± 3.16	92.54	4.67	**< 0.001**
sex	13.60 ± 3.95	70.80	3.44	**< 0.001**
**C. wintering area longitude**^**a**^				
Intercept	-11.97 ± 1.17	1.17	69.83	**< 0.001**
year_2017/18	0.07 ± 0.13	0.13	70.01	0.555
year_2018/19	0.02 ± 0.12	0.12	70.06	0.875
year_2019/20	0.02 ± 0.13	0.13	70.12	0.131
sex	0.17 ± 0.57	0.57	78.94	0.769
**D. wintering area latitude**^**a**^				
Intercept	13.94 ± 1.55	92.06	9.02	**< 0.001**
year_2017/18	− 1.36 ± 0.46	70.11	− 2.96	**0.004**
year_2018/19	− 0.41 ± 0.45	70.49	− 0.90	0.370
year_2019/20	− 1.19 ± 0.48	71.06	− 3.99	**< 0.001**
sex	− 1.18 ± 1.68	130.31	− 0.70	0.480
**E. departure from wintering area (days)**^**b**^				
Intercept	85.41 ± 3.70	101.82	23.11	**< 0.001**
year_2017/18	3.93 ± 3.61	57.57	1.09	0.281
year_2018/19	3.01 ± 4.29	70.68	0.70	0.485
year_2019/20	− 1.92 ± 3.52	74.29	− 0.55	0.587
sex	5.59 ± 3.66	63.53	1.53	0.131
**F. arrival at colony (days)**^**c**^				
Intercept	105.06 ± 1.82	99.03	57.86	**< 0.001**
year_2017/18	4.44 ± 1.45	45.87	3.07	**0.004**
year_2018/19	5.81 ± 1.85	54.09	3.14	**0.003**
year_2019/20	6.08 ± 1.48	55.07	4.11	**< 0.001**
sex	1.38 ± 2.04	69.22	0.68	0.501

Between-year variation in the dependent variables was accounted for by adding year as a fixed effect to the models (2016/2017 as a reference). P-values ≤ 0.05 are presented in bold

^a^n = 138 tracks of 64 individuals (29 ♂ + 35 ♀)

^b^n = 107 tracks of 60 individuals (27 ♂ + 33 ♀)

^c^n = 105 tracks of 60 individuals (27 ♂ + 33 ♀)

Birds arrived at their wintering areas on 18 September (range 5 September–15 November), with females arriving earlier than males (Table 1, pt. B). These wintering areas were mainly located at (i) the west coast of West Africa, (ii) the south coast of West Africa and (iii) the coast of Namibia and South Africa (Fig. 1; see also Additional file 1: Table S7).

The total autumn migration distance was 5281 km (range 3974–11,027). This distance was covered in 12 days (range 2–67) with a total speed of 602 km/day (range 81–2231). The actual duration of the period in which birds covered the distance was 7 days (range 2–16), the travel speed 742 km/day (range 323–2231).

Most birds used a single wintering area in which they stayed for 192 days (range 116–247; n = 103 tracks of 58 individuals). Four birds showed an exception to this pattern in some years by using two wintering areas (distance: 877 km; range 616–1328; n = 5 tracks of 4 individuals). For the subset of birds for which we could also quantify the length of their stay at their multiple wintering areas, this was 101 days (range 63–130; n = 4 tracks of 3 individuals) for the first and 105 days (range 66–146; n = 3 tracks of 2 individuals) for the second wintering area. With respect to the wintering distribution, we found no significant difference between the sexes (Table 1, pt. C and D). Furthermore, while some pair members overwintered in the same wintering area (n = 3 couples), others went to different wintering areas (n = 4 couples).

Birds of both sexes left their wintering areas on 29 March (range 10 February–25 May) (Table 1, pt. E). They travelled a total distance of 5463 km (range 3497–11,447) to their breeding colony, i.e. further than during autumn migration (Table 2, pt. A). This distance was covered in 22 days (range 4–99) with a total speed of 358 km/day (range 61–1277), such that birds travelled longer and more slowly in spring than in autumn (Table 2, pt. B and D). Similarly, the actual duration of spring migration, at 9 days (range 4–23), was longer than that in autumn, and covered with a lower travel speed of 560 km/day (range 209–1277) (Table 2, pt. C and E).

**Table 2:** Summary of models testing for effects of season (spring as a reference) and sex (female as a reference) on (A) total migration distance, (B) total duration of migration, (C) actual duration of migration, (D) total speed of migration and (E) travel speed of common terns deployed with geolocators during 2016–2020

**Model**	estimate ± SE*	df	t-value	p-value
**A. total migration distance (km)**^**a**^				
Intercept	8.51 ± 0.04	102.20	237.88	**< 0.001**
season	0.03 ± 0.01	174.30	3.19	**0.002**
year_2017/18	0.03 ± 0.01	175.60	2.08	**0.039**
year_2018/19	0.01 ± 0.01	178.10	0.55	0.583
year_2019/20	0.05 ± 0.01	180.00	3.64	**< 0.001**
sex	0.01 ± 0.04	167.10	0.18	0.859
**B. total duration of migration (days)**^**a**^				
Intercept	2.16 ± 0.13	169.59	17.16	**< 0.001**
season	0.55 ± 0.07	182.36	8.31	**< 0.001**
year_2017/18	0.08 ± 0.11	195.17	0.74	0.460
year_2018/19	0.05 ± 0.11	210.31	0.47	0.643
year_2019/20	0.27 ± 0.11	223.66	2.47	**0.014**
sex	− 0.04 ± 0.13	69.97	− 0.33	0.741
**C. actual duration of migration (days)**^**b**^				
Intercept	1.82 ± 0.12	96.30	17.14	**< 0.001**
season	0.28 ± 0.09	106.84	3.21	**0.001**
year_2017/18	− 0.09 ± 0.11	103.66	− 0.75	0.454
year_2018/19	− 0.05 ± 0.12	110.90	− 0.42	0.673
year_2019/20	0.09 ± 0.11	113.67	0.85	0.396
sex	0.16 ± 0.08	38.13	1.92	0.063
**D. total speed of migration (km/day)**^**a**^				
Intercept	6.38 ± 0.10	183.03	61.82	**< 0.001**
season	− 0.52 ± 0.06	187.88	− 8.38	**< 0.001**
year_2017/18	− 0.05 ± 0.10	206.74	− 0.47	0.637
year_2018/19	− 0.06 ± 0.11	225.32	− 0.60	0.547
year_2019/20	− 0.22 ± 0.10	235.23	− 2.27	**0.024**
sex	0.01 ± 0.10	63.16	0.12	0.902
**E. travel speed (km/day)**^**b**^				
Intercept	6.59 ± 0.10	89.08	68.91	**< 0.001**
season	− 0.29 ± 0.08	112.05	− 3.51	**< 0.001**
year_2017/18	0.10 ± 0.11	110.38	0.94	0.351
year_2018/19	0.05 ± 0.11	113.81	0.41	0.680
year_2019/20	− 0.06 ± 0.10	110.34	− 0.62	0.536
sex	− 0.12 ± 0.07	34.55	− 1.80	0.080

Between-year variation in the dependent variables was accounted for by adding year as a fixed effect to the models (2016/2017 as a reference). P-values ≤ 0.05 are presented in bold

^a^n = 243 (138 + 105) tracks of 64 individuals

^b^n = 120 (89 + 31) tracks of 47 individuals

^*^Data were log10-transformed

In 74 tracks of 48 individuals, 1–4 spring stopover sites were used at the coasts of Mauretania, Western Sahara, Morocco or Portugal, at the Canary Islands or in the Bay of Biscay (see Additional file 1: Table S6). The number of stopover sites did not differ between spring and autumn migration (0.25 ± 0.16 SE, z = 1.56, *p* = 0.119; n = 123 tracks of 53 individuals), but the probability that birds used stopover sites was higher in spring than in autumn (2.24 ± 0.42 SE, z = 5.35, *p* < 0.001; n = 243 tracks of 64 individuals).

Spring migration ended on 20 April (range 1 April–8 May), and arrival at the breeding colony did not differ between the sexes (Table 1, pt. F).

### Repeatability of the spatiotemporal distribution

**Fig. 2:**
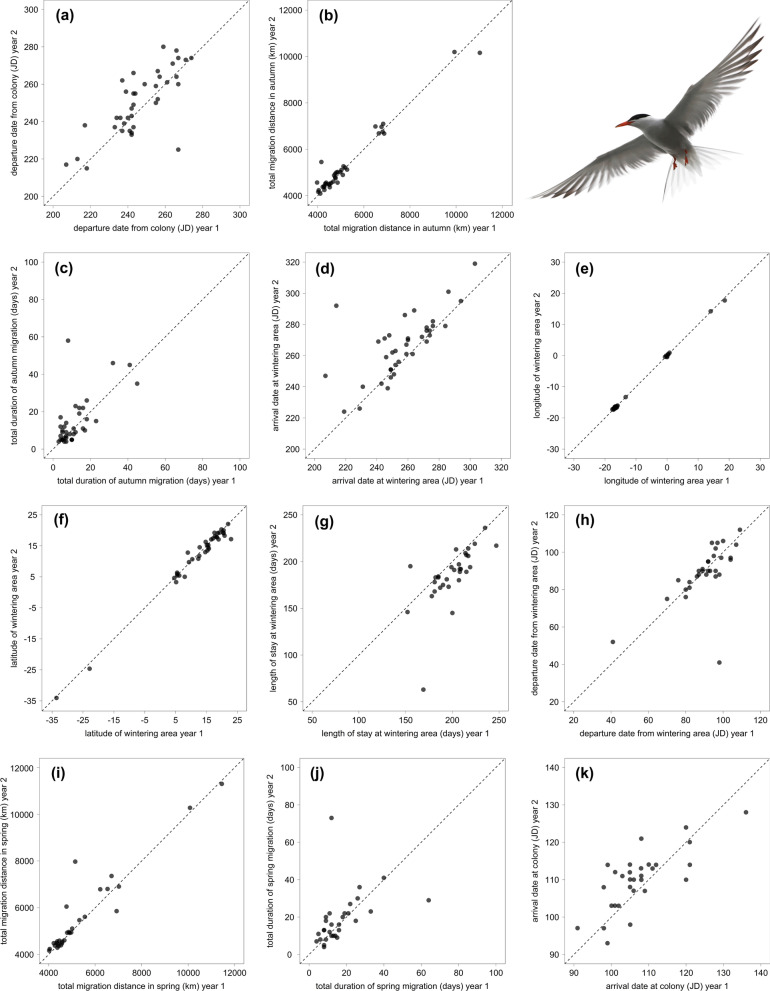
Repeatability of the **a** departure date from the colony, **b** total migration distance in autumn, **c** total duration of autumn migration, **d** arrival date at the wintering area, **e** longitude of the wintering area, **f** latitude of the wintering area, **g** length of stay at the wintering area, **h** departure date from the wintering area, **i** total migration distance in spring, **j** total duration of spring migration and **k** arrival date at the colony. For the purpose of visualisation, we randomly selected 2 years of data per individual and plotted them against each other (a–f: n = 38; g–h: n = 31; i–k: n = 30). All models were, however, run using all data available for each individual. Dotted lines represent lines of equality. JD, Julian Day

Both the annual departure date from the breeding colony and arrival date at the wintering area were highly repeatable (R = 0.72, *p* < 0.001 and R = 0.63, *p* < 0.001; Fig. 2a, d; Table 3, pt. A), such that the total duration of autumn migration was highly repeatable too (R = 0.66, *p* < 0.001; Fig. 2c; Table 3, pt. A). During autumn migration, most birds (78%) showed consistent stopover behaviour, with 22 birds stopping in each of their 60 tracks and 9 birds never stopping in any of their 27 tracks. Only 9 birds were inconsistent in whether or not they stopped in their 27 tracks.

**Table 3:** Repeatability (R) of the distance, phenology and spatial distribution of common tern migration, with and without including year as a fixed effect in the models. Note that all models included sex as a fixed effect

Trait	A. sex- & year-specific repeatability			B. sex-specific repeatability		
	R ± SE	95% CI	p-value	R ± SE	95% CI	p-value
**migration distance**						
total migration distance in autumn^a^	0.937 ± 0.015	0.906–0.963	**< 0.001**	0.934 ± 0.017	0.895–0.959	**< 0.001**
total migration distance in spring^c^	0.925 ± 0.021	0.881–0.961	**< 0.001**	0.907 ± 0.025	0.851–0.947	**< 0.001**
**phenology**						
departure date from colony^a^	0.719 ± 0.063	0.579–0.827	**< 0.001**	0.571 ± 0.081	0.391–0.717	**< 0.001**
arrival date at wintering area^a^	0.630 ± 0.080	0.473–0.764	**< 0.001**	0.533 ± 0.090	0.318–0.692	**< 0.001**
total duration of autumn migration^a^	0.661 ± 0.072	0.503–0.795	**< 0.001**	0.630 ± 0.080	0.448–0.759	**< 0.001**
length of stay at wintering area^b^	0.638 ± 0.089	0.451–0.796	**< 0.001**	0.598 ± 0.095	0.392–0.751	**< 0.001**
departure date from wintering area^b^	0.358 ± 0.130	0.100–0.618	**< 0.001**	0.368 ± 0.130	0.084–0.611	**< 0.001**
arrival date at colony^c^	0.745 ± 0.064	0.617–0.872	**< 0.001**	0.713 ± 0.075	0.550–0.837	**< 0.001**
total duration of spring migration^c^	0.646 ± 0.085	0.492–0.804	**< 0.001**	0.662 ± 0.085	0.475–0.806	**< 0.001**
**spatial distribution**						
wintering area longitude^a^	0.998 ± 0.001	0.997–0.999	**< 0.001**	0.998 ± 0.001	0.997–0.999	**< 0.001**
wintering area latitude^a^	0.979 ± 0.005	0.970–0.988	**< 0.001**	0.973 ± 0.007	0.958–0.983	**< 0.001**

P-values ≤ 0.05 are presented in bold

^a^n = 138 tracks of 64 individuals

^b^n = 107 tracks of 60 individuals

^c^n = 105 tracks of 60 individuals

**Fig. 3:**
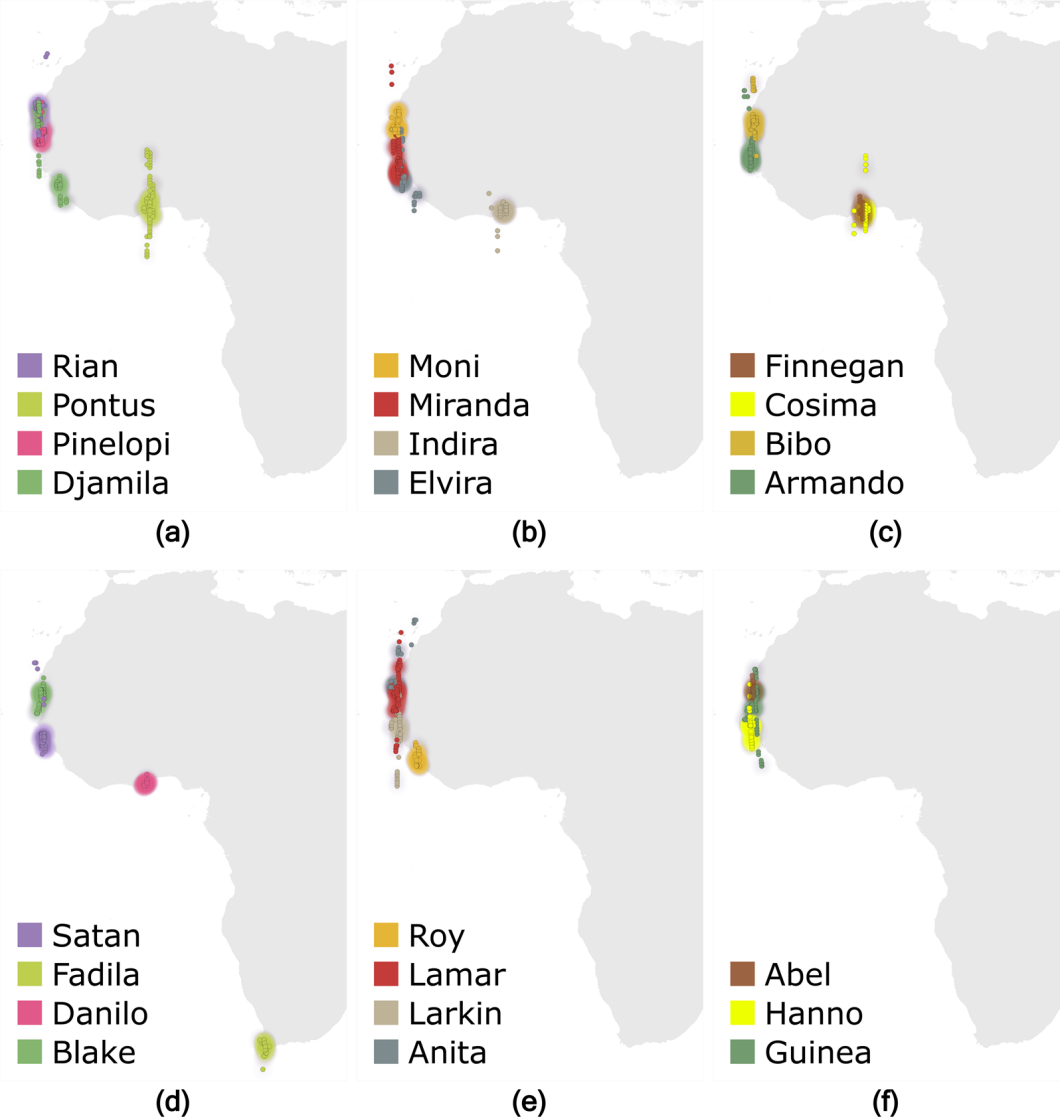
Wintering areas of common terns tracked with light-level geolocators for four (**a**–**c**, n = 12) or three years (**d**–**f**, n = 11). Dots represent an individual’s locations (estimated using FLightR) at daily twilights from one week after the estimated arrival up to one week before the estimated departure from the wintering area. For the birds of which the geolocator stopped working at the wintering area, data for that year were used until the last estimated position. The heatmaps of the twilight positions were produced in QGIS using quartic kernel density with a 2º radius, and visually scaled from 1 (transparent) to 300 (most intense) twilights

The total migration distance (R = 0.94 *p* < 0.001; Fig. 2b; Table 3, pt. A), and longitude and latitude of the wintering areas were highly repeatable (R = 1.00, *p* < 0.001 and R = 0.98, *p* < 0.001; Fig. 2e, f; Fig. 3a–f; Table 3, pt. A), although the individual migration routes showed variability between years (see Additional file 1: Fig. S1). The individual length of stay at the wintering area was highly repeatable too (R = 0.64, *p* < 0.001; Fig. 2g; Table 3, pt. A).

The timing of spring migration was moderately repeatable with respect to the departure from the wintering area (R = 0.36, *p* < 0.001; Fig. 2h; Table 3, pt. A), although arrival date at the breeding colony and total duration of spring migration were again highly repeatable (R = 0.75, *p* < 0.001 and R = 0.65, *p* < 0.001; Fig. 2k, j; Table 3, pt. A). As in autumn, the individual total spring migration distance was highly repeatable (R = 0.93, *p* < 0.001; Fig. 2i; Table 3, pt. A). Spring stopover behaviour was consistent in 63% of birds, since 6 birds stopped in each of their 13 tracks, 13 birds never stopped in any of their 33 tracks, and 11 birds were inconsistent in whether they stopped or not (29 tracks).

Removing year as a fixed effect from the models led to minor or no reductions in the repeatability estimates for migration distance, the spatial distribution of the wintering areas or the phenology of spring migration, but to somewhat stronger reductions of the repeatability estimates for the phenology of autumn migration (Table 3, pt. B).

## Discussion

Understanding the evolution of migration requires knowledge of the patterns, sources, and consequences of variation in migratory behaviour. Moreover, since many populations of migratory species show rapid population declines (e.g. [6]), with populations of long-distance migrants declining faster than those of short-distance migrants, at least among birds (e.g. [70]), this knowledge also is crucial for facilitating implementation of knowledge-based conservation measures (e.g. [7, 8]). As a first step towards understanding the causes and consequences of variation in migratory behaviour of a locally endangered species, the common tern, we tracked 138 migratory journeys undertaken by 64 individual birds. We used these data to (i) identify the annual spatiotemporal distribution of these birds, (ii) show the individual repeatability of their migratory behaviour to be at the high end of the range reported for birds, and (iii) demonstrate that only the repeatability of the phenology of autumn migration is substantially affected by inter-annual variation in migratory behaviour across the population.

### Spatiotemporal distribution

The common terns from our study population in northwest Germany migrated along the East Atlantic Flyway. In autumn, females departed from the breeding colony, and arrived at the wintering areas ca. two weeks before males did – a pattern referred to as protogyny [71]. While the opposite pattern, protandry, seems to be the norm for autumn migration in birds [26], protogyny has previously been observed in common terns ([72–75, but see 53]), as well as in other migratory seabirds (e.g. [25, 76]), raptors (e.g. [77]), and songbirds (e.g. [71]). In common terns, it may be explained by males performing longer post-fledging parental care than females [72], a pattern that is interesting in itself, as it may lead to offspring traits developing during this phase to be more strongly influenced by the father. An example may have recently been found in oystercatchers (*Haematopus ostralegus*), in which fledglings socially inherit their migratory behaviour from their fathers [78].

We found common terns to migrate both over-sea and across-land (Fig. 1). Similar across-land migration was recently reported in tracking studies on various sea- and shorebird species, among which arctic terns (*Sterna paradisaea*) [61], lesser black-backed gulls (*Larus fuscus*) [79], reddish egrets (*Egretta rufescens*) [80], brown pelicans (*Pelecanus occidentalis*) [80], red knots (*Calidris canutus*) [80–82], as well as common terns [75]. As such, our results add to the growing evidence for the hypothesis that across-land migration is a much more widespread migration strategy for waterbirds than previously thought, which may have important implications for the protection of these birds [80].

Some terns were found to use stopover sites at the coasts and seas of Morocco, Western Sahara, Mauritania, and the Canary Islands (see Additional file 1: Table S6), hence showing similar stopover behaviour to many other seabird species [83]. Most birds (76%) then wintered along the coast of West Africa, but some (19%) wintered further south or even as far south as Namibia and South Africa (5%) (Fig. 1). These regions are located in three different Large Marine Ecosystems: the Canary Current Large Marine Ecosystem in the west of West Africa, the Guinea Current Large Marine Ecosystem in the south of West Africa, and the Benguela Current Large Marine Ecosystem in Namibia and South Africa. These ecosystems are characterised by strong upwelling and show high rates of primary productivity (e.g. [84, 85]), thereby providing abundant food (e.g. [83, 86–88]) and being of extreme importance for a wide range of marine animals [89]. These ecosystems, however, are known to be affected by current climate change (for a review see [90]), such that consequences for primary and fish production are expected [91]. Such consequences, in turn, are likely to affect many marine animals (for a review see [92]), including seabirds (e.g. [93, 94]).

On the return journey to the breeding colony in spring, the terns showed a higher probability to use stopover sites, although the number of stopover sites used did not differ from that in autumn. We also found the total duration of migration to be longer in spring than in autumn. This pattern corresponds with that found in roseate terns (*Sterna dougallii*) wintering in West-Africa [88], but contrasts with the more general pattern of avian migration being faster in spring than in autumn [95, 96]. Perhaps the mating process [97] and benefit of a short interval between arrival and egg laying to facilitate early breeding [98] exert a stronger selection pressure on the terns’ condition at arrival in the breeding area than on their arrival date per se, leading them to spend more time foraging during spring than autumn migration. Alternatively, feeding conditions may be better at the breeding than wintering area, such that there is less need to forage during autumn than spring migration. Or perhaps the pattern can be explained by the prevailing wind conditions during migration. Winds rotate clockwise in the North Atlantic, thereby offering tailwind in autumn, but headwind in spring [99], potentially leading terns, who are very susceptible to wind [100], to fly more slowly in spring than in autumn. Effects of other environmental conditions that differ consistently between the seasons, such as temperature or rainfall, could pose an additional explanation [101, 102] and future work should investigate whether and how such environmental factors influence the various aspects of migratory behaviour.

### Repeatability of the spatiotemporal distribution

We found an extremely high repeatability in common tern wintering area locations. These birds thus are highly site-faithful to both their breeding colony and individual wintering areas. Similar observations have been made in various other migratory bird species (e.g. [33, 103]), suggesting many birds to generally use a genetically inherited (see [104]) or familiar (previously encountered or socially learned) wintering area. The latter may be facilitated by spatial memory formation [105], develop through ontogeny as birds change from exploratory to more consistent migratory behaviour (e.g. [106, 107]), and come with the benefit of increased local knowledge (e.g. [108]). On the other hand, ‘simply’ returning to a familiar location could also pose a risk if conditions at a location were to become unsuitable but birds remained site-faithful (see [13]).

In our case, more than 75% of the terns we tracked wintered along the west coast of West Africa. A large proportion of our study population thus appears to be dependent on a relatively small geographic (wintering) region. Given this region is predicted to become less suitable with further change of the environmental conditions (for climate predictions see [109]; for fish stock predictions see [91]), if these birds do not change wintering area, we may expect them to start performing increasingly poorly, i.e. to survive less well, or to show reduced breeding performance in the case of carry-over effects to the breeding season (e.g. [110, 111]). This, in turn, could lead to (i) a local population decline if birds with another migratory phenotype (i.e. birds wintering in the other regions) are not able to compensate for such a decrease in performance, or even to (ii) a general population decline if terns from other populations use the same wintering areas [112, 113], if no new suitable wintering areas emerge, or other existing wintering areas decline in suitability too, or if other existing wintering areas cannot sustain an increasing number of birds (i.e. if there is a lack of alternatives [114]).

Contrary to the high individual repeatability in wintering area, we found migration routes to be more inconsistent (see Additional file 1: Fig. S1). Route inconsistency has been demonstrated for various migratory seabirds (e.g. [115]), shorebirds (e.g. [116]) and raptors (e.g. [117]), as well as songbirds (e.g. [118]), and suggests migration to be influenced by variable or unpredictable environmental conditions experienced *en route* (e.g. [119, 120]). Interestingly, in our study, the observed inconsistency in migration route did not result in a low repeatability of the total migration time. This suggests that the birds either managed to find what they needed by adjusting their routes to the environmental conditions, or that they prioritised their time keeping over finding what they needed. In the latter case, we would expect environmental conditions experienced *en route* to have carry-over effects on body condition at arrival [121], which first (population-level) analyses of the study colony did not support [122], but which warrants further investigation.

In terms of phenology, departure date from the colony, arrival date at the wintering area, and arrival date at the breeding colony were highly repeatable. While consistency in the timing of migration has been demonstrated for several migratory bird species [32], the degree of repeatability in our study is at the high end of the reported spectrum. Such strong individual consistency may partly result from a genetic basis to migration phenology [123]. For one of the traits, arrival date at the breeding colony, quantitative genetic analyses of our long-term dataset (7436 first annual registrations of 1630 individuals across 1994–2019) indeed indicate an additive genetic component of c. 20% (Moiron et al. unpublished). Any non-genetic part of the repeatability in phenology should reflect what quantitative geneticists refer to as ‘permanent environment or permanent individual effects’, i.e. non-genetic stable differences between individuals for example resulting from (i) life-long carry-over effects of natal conditions (e.g. hatching date [5, 124]) or (ii) stochasticity (e.g. initial social and environmental effects) having turned into routine via reinforcement through memory models (see [105] for a spatial context, and [125] for the role of stochasticity in determining breeding phenology).

Interestingly, the repeatability of the departure date from the wintering area was only moderate repeatable and half that of the arrival date at the breeding colony (with the 95% CI overlapping only 0.1%). As such, birds relatively inconsistently timed their departure for spring migration, but then sped up or slowed down to arrive at the colony at their individual-specific date. The additional fact that the repeatability of spring arrival date itself was largely independent of general inter-annual variation (since the repeatability with or without accounting for year as a fixed effect differed by only 3.2%; Table 2) suggests there may be adjustment to keep an internal clock (synchronisation, [123]) or that a late departure, for example, is associated with more favourable conditions *en route*.

When assessing whether our estimates of the repeatability of migratory traits other than arrival date at the breeding colony were affected by general inter-annual variation, we found only the repeatability of autumn migration phenology (i.e. departure from the breeding colony and arrival at the wintering area) to be increased when correcting for year (by 15 and 10%, respectively). This increase suggests that the (tagged) population as a whole departs and arrives substantially earlier or later in some years, while the phenological ranking of individual birds remains largely the same. A possible mechanism underlying this inter-annual population variation in autumn migration could be annual variation in breeding success, which indeed is substantial in the study population [126]. In years with poor herring abundance, common terns show strong brood reduction [127, 128]. As a result, most birds can perhaps depart (and therefore arrive) earlier in years of relatively unsuccessful reproduction, such as observed in other species (e.g. [129, 130]). Alternatively, birds may all display largely similar reaction norms to drivers of autumn migration, or there may exist a strong social influence on departure [131].

## Conclusions

Using a multi-year individual-based dataset of migrating common terns, we showed these birds to display remarkable within-individual consistency in their migratory behaviour, with repeatability values ranging from 0.36 for the departure date from the wintering area to 1.00 (longitude) and 0.98 (latitude) for the wintering area. This knowledge can be used to (i) learn whether migratory behaviour is underpinned by (epi)genetic heritability, developmental plasticity or phenotypic flexibility, (ii) assess the consequences of migratory behaviour at different timescales, and (iii) use spatiotemporal knowledge of risks individuals or populations may face to inform or improve conservation management. Hence, our study provides essential knowledge (also for future work), and exemplifies how intensive individual-based tracking studies can improve our understanding of migratory behaviour.

## Supplementary Information

**Additional file 1**. Tables S1–S7 and Figure S1.

**Additional file 2**. R code for the analysis of our light-level geolocator data.

## Data Availability

The datasets underlying the results and conclusions reported in this article are provided in Additional file 1; the R code for the analysis of light-level data in Additional file 2. Light-level data are available upon request to the corresponding author.
