# Correction: High individual repeatability of the migratory behaviour of a long-distance migratory seabird

**DOI:** 10.1186/s40462-023-00369-2

**Published:** 2023-01-26

**Authors:** Nathalie Kürten, Heiko Schmaljohann, Coraline Bichet, Birgen Haest, Oscar Vedder, Jacob González‑Solís, Sandra Bouwhuis

**Affiliations:** 1 Institute of Avian Research, An Der Vogelwarte 21, 26386 Wilhelmshaven, Germany; 2 Institute of Biology and Environmental Sciences, University of Oldenburg, Carl‑von‑Ossietzky‑Str. 9–11, 26129 Oldenburg, Germany; 3 Centre d’Etudes Biologiques de Chizé, UMR 7372, CNRS-Université de La Rochelle, 79360 Villiers‑en‑Bois, France; 4 Department of Bird Migration, Swiss Ornithological Institute, 6204 Sempach, Switzerland; 5 Institut de Recerca de La Biodiversitat and Departament de Biologia Evolutiva, Ecologia I Ciències Ambientals, Universitat de Barcelona, Av. Diagonal 643, 08028 Barcelona, Spain

**Correction to** **:** **Movement Ecology (2022) 10:5** **https://doi.org/10.1186/s40462-022-00303-y** **.**

Following publication of the original article [1], the authors identified errors in the reported ranges of arrival dates to, and departure dates from, the breeding colony and wintering areas. These errors neither had an effect on any of the analyses or other values reported in the results section, nor on the conclusions drawn in the publication. The authors apologise to the readers for the inconvenience.

Corrections (marked in **bold**):

1. With respect to the average spatiotemporal distribution, common terns left the breeding colony in the northwest of Germany on 5 September (range 24 July–**7** October), with females leaving earlier than males (Table 1, pt. A).

2. Birds arrived at their wintering areas on 18 September (range **26 July**–15 November), with females arriving earlier than males (Table 1, pt. B).

3. Birds of both sexes left their wintering areas on 29 March (range **14 January**–25 **April**) (Table 1, pt. E).

4. Spring migration ended on 20 April (range 1 April–**16** May), and arrival at the breeding colony did not differ between the sexes (Table 1, pt. F).

The original article [1] has been corrected.
