# Supplementary material for: High individual repeatability of the migratory behaviour of a long-distance migratory seabird
Source: Mov Ecol. 2022 Feb 5;10:5. doi: 10.1186/s40462-022-00303-y (PMC8817581; doi:10.1186/s40462-022-00303-y)
Supplement: Supplementary file 1 — Additional file 1. Tables S1–S7 and Figure S1. [file 40462_2022_303_MOESM1_ESM.docx]

Additional file 1

for

**High individual consistency in the migratory behaviour of a long-distance migratory seabird**

by

Nathalie Kürten, Heiko Schmaljohann, Coraline Bichet, Birgen Haest, Oscar Vedder, Jacob González-Solís, Sandra Bouwhuis

**Table S1** Information on common terns deployed with light-level geolocators in 2016, 2017, 2018 and/or 2019: name, sex, age at first deployment, annual geolocator number and data availability, and sum of tracks. An “x” indicates the absence of available data due to the bird not returning, not reproducing, or losing its geolocator (notes).

| **name** | **sex** | **age at**  **1st dep** | **geo 16/17** | **data 16/17** | **geo 17/18** | **data 17/18** | **geo 18/19** | **data 18/19** | **geo 19/20** | **data 19/20** | **sum of tracks** | **notes** |
| --- | --- | --- | --- | --- | --- | --- | --- | --- | --- | --- | --- | --- |
| Elvira | f | 15 | Z436_16 | full | BD664_17 | full | BL133_18 | till 10.04 | BS701_19 | full | 4 |  |
| SirDavid | m | 22 | Z435_16 | full | BD274_17 | x | ‒ | ‒ | ‒ | ‒ | 1 | not returned 2018 |
| Moni | f | 10 | Z445_16 | full | BD277_17 | full | BL143_18 | till 26.10 | BS721_19 | full | 4 |  |
| Auguste | f | 22 | Z441_16 | till 09.01 | BD275_17 | x | ‒ | ‒ | ‒ | ‒ | 1 | not returned 2018 |
| Larkin | m | 11 | Z440_16 | full | BD278_17 | till 26.01 | BL139_18 | till 19.10 | BS712_19 | x | 3 | no rep 2020 |
| Armando | m | 16 | Z447_16 | full | BD276_17 | full | BL156_18 | till 02.11 | BS733_19 | full | 4 |  |
| Pinelopi | f | 6 | Z446_16 | full | BD669_17 | full | BL131_18 | till 05.10 | BS709_19 | full | 4 |  |
| Roy | m | 15 | Z437_16 | till 23.01 | BD665_17 | full | BL128_18 | till 11.10 | BS705_19 | full | 4 |  |
| Danilo | m | 12 | Z449_16 | full | BD668_17 | full | BL153_18 | till 02.04 | BT692_19 | x | 3 | not returned 2020 |
| Abel | m | 8 | Z444_16 | full | BD666_17 | full | BL127_18 | till 27.10 | BS706_19 | no data | 3 |  |
| Miranda | f | 10 | Z453_16 | full | BD667_17 | full | BL147_18 | till 19.01 | BT689_19 | full | 4 |  |
| Anita | f | 16 | Z450_16 | full | BD670_17 | full | BL161_18 | till 21.11 | BS723_19 | x | 3 | not returned 2020 |
| Indira | f | 6 | Z438_16 | full | BD671_17 | full | BL135_18 | full | BS711_19 | full | 4 |  |
| Bibo | m | 13 | Z594_16 | full | BD672_17 | full | BL132_18 | till 05.12 | BS715_19 | full | 4 |  |
| Djamila | f | 6 | Z448_16 | full | BD673_17 | full | BD679_18 | full | BS728_19 | full | 4 |  |
| Bea | f | 17 | Z452_16 | full | BD675_17 | till 30.12 | BL130_18 | x | ‒ | ‒ | 2 | not returned 2019 |
| Finnegan | m | 6 | Z451_16 | full | BD676_17 | full | BL146_18 | full | BS722_19 | full | 4 |  |
| Lamar | m | 6 | Z595_16 | full | BD678_17 | full | BL157_18 | till 03.04 | ‒ | ‒ | 3 |  |
| Jantje | f | 6 | Z596_16 | full | ‒ | ‒ | ‒ | ‒ | BS713_19 | full | 2 |  |
| Cosima | f | 6 | Z443_16 | full | BD680_17 | full | BL158_18 | till 10.10 | BS708_19 | till 09.01 | 4 |  |
| Rian | m | 13 | Z434_16 | full | BD679_17 | full | BL144_18 | full | BS140_19 | full | 4 |  |
| Pontus | m | 5 | Z597_16 | till 19.03 | Z452_17 | till 14.02 | BL163_18 | till 14.02 | BS718_19 | full | 4 |  |
| Elijah | m | 8 | Z439_16 | x | ‒ | ‒ | ‒ | ‒ | ‒ | ‒ | 0 | not returned 2017 |
| Belgus | m | 11^a^ | Z442_16 | x | ‒ | ‒ | ‒ | ‒ | ‒ | ‒ | 0 | no rep 2017 |
| Lulu | f | 18 | ‒ | ‒ | Z436_17 | full | BL126_18 | full | ‒ | ‒ | 2 |  |
| Nikolas | m | 7 | ‒ | ‒ | Z438_17 | no data | BL154_18 | till 18.12 | BU573_19 | full | 3 |  |
| Fadila | f | 5 | ‒ | ‒ | Z440_17 | full | BL150_18 | till 13.11 | BS727_19 | full | 3 |  |
| Thora | f | 3 | ‒ | ‒ | Z443_17 | x | ‒ | ‒ | ‒ | ‒ | 0 | geo lost 2018 |
| Raimundo | m | 17 | ‒ | ‒ | Z444_17 | x | ‒ | ‒ | ‒ | ‒ | 0 | no rep 2018 & 2019 |
| Blake | m | 8 | ‒ | ‒ | Z446_17 | full | BL145_18 | till 22.12 | BS714_19 | full | 3 |  |
| Joachim | m | 18 | ‒ | ‒ | Z447_17 | full | BL129_18 | till 06.11 |  |  | 2 |  |
| Guinea | f | 7 | ‒ | ‒ | Z449_17 | full | BL149_18 | till 01.11 | BS716_19 | full | 3 |  |
| Henrike | f | 3 | ‒ | ‒ | Z450_17 | x | ‒ | ‒ | ‒ | ‒ | 0 | not returned 2018 |
| Melek | f | 3 | ‒ | ‒ | Z451_17 | full | BD670_18 | full | BS732_19 | x | 2 | not returned 2020 |
| Satan | m | 6 | ‒ | ‒ | Z435_17 | full | BL138_18 | till 28.01 | BS740_!9 | full | 3 |  |
| Hanno | m | 4 | ‒ | ‒ | Z453_17 | full | BD277_18 | full | BS717_19 | full | 3 |  |
| Desiree | f | 21 | ‒ | ‒ | Z594_17 | x | ‒ | ‒ | ‒ | ‒ | 0 | not returned 2018 |
| Nanny | f | 10 | ‒ | ‒ | Z596_17 | x | ‒ | ‒ | ‒ | ‒ | 0 | not returned 2018 |
| Arno | m | 4 | ‒ | ‒ | Z434_17 | full | BD276_18 | no data | BS719_19 | x | 1 | not returned 2020 |
| Antoine | m | 5 | ‒ | ‒ | ‒ | ‒ | BD668_18 | full | BS731_19 | full | 2 |  |
| Henna | f | 17 | ‒ | ‒ | ‒ | ‒ | BL148_18 | x | ‒ | ‒ | 0 | not returned 2019 |
| Katja | f | 23 | ‒ | ‒ | ‒ | ‒ | BL136_18 | x | ‒ | ‒ | 0 | not returned 2019 |
| London | m | 8 | ‒ | ‒ | ‒ | ‒ | BD665_18 | full | BS729_19 | full | 2 |  |
| BigBoss | m | 10 | ‒ | ‒ | ‒ | ‒ | BL159_18 | x | ‒ | ‒ | 0 | not returned 2019 |
| Aristide | m | 8 | ‒ | ‒ | ‒ | ‒ | BL155_18 | till 10.01 | ‒ | ‒ | 1 |  |
| Martha | f | 19 | ‒ | ‒ | ‒ | ‒ | BL162_18 | full | ‒ | ‒ | 1 |  |
| Sarina | f | 10 | ‒ | ‒ | ‒ | ‒ | BD667_18 | full | BS710_19 | full | 2 |  |
| Lucius | m | 17 | ‒ | ‒ | ‒ | ‒ | BL164_18 | full | ‒ | ‒ | 1 |  |
| Princess | f | 10 | ‒ | ‒ | ‒ | ‒ | BD672_18 | full | BS703_19 | full | 2 |  |
| Nathan | m | 5 | ‒ | ‒ | ‒ | ‒ | BD669_18 | x | ‒ | ‒ | 0 | lost geo 2019 |
| Padme | f | 15 | ‒ | ‒ | ‒ | ‒ | BD678_18 | full | BU571_19 | till 03.04 | 2 |  |
| Carmela | f | 19 | ‒ | ‒ | ‒ | ‒ | BL140_18 | x | ‒ | ‒ | 0 | not returned 2019 |
| Beccy | f | 15 | ‒ | ‒ | ‒ | ‒ | BL141_18 | full | BS702_19 | full | 2 |  |
| Yealle | f | 10 | ‒ | ‒ | ‒ | ‒ | BL125_18 | till 11.10 | BS704_19 | full | 2 |  |
| Gump | m | 15 | ‒ | ‒ | ‒ | ‒ | BL160_18 | x | ‒ | ‒ | 0 | not returned 2019 |
| Pius | m | 7 | ‒ | ‒ | ‒ | ‒ | BL151_18 | x | ‒ | ‒ | 0 | no rep 2019 |
| Hirundo | m | 6 | ‒ | ‒ | ‒ | ‒ | BL152_18 | full | BL126_19 | full | 2 |  |
| Benno | m | 17 | ‒ | ‒ | ‒ | ‒ | BL142_18 | no data | ‒ | ‒ | 0 |  |
| Puck | m | 18 | ‒ | ‒ | ‒ | ‒ | BL137_18 | till 25.03 | ‒ | ‒ | 1 |  |
| Kirk | m | 8 | ‒ | ‒ | ‒ | ‒ | BL134_18 | till 16.10 | BS707_19 | full | 2 |  |
| Bofur | m | 6 | ‒ | ‒ | ‒ | ‒ | ‒ | ‒ | BS724_19 | full | 1 |  |
| Lineka | f | 5 | ‒ | ‒ | ‒ | ‒ | ‒ | ‒ | BS726_19 | full | 1 |  |
| Maigold | f | 5 | ‒ | ‒ | ‒ | ‒ | ‒ | ‒ | BS725_19 | full | 1 |  |
| Autumn | f | 6 | ‒ | ‒ | ‒ | ‒ | ‒ | ‒ | BS738_19 | full | 1 |  |
| Primrose | f | 4 | ‒ | ‒ | ‒ | ‒ | ‒ | ‒ | BS734_19 | till 27.11 | 1 |  |
| Russell | m | 8 | ‒ | ‒ | ‒ | ‒ | ‒ | ‒ | BS730_19 | full | 1 |  |
| Cupido | m | 5 | ‒ | ‒ | ‒ | ‒ | ‒ | ‒ | BS735_19 | full | 1 |  |
| Ribanna | f | 6 | ‒ | ‒ | ‒ | ‒ | ‒ | ‒ | BS736_19 | full | 1 |  |
| Luca | m | 9 | ‒ | ‒ | ‒ | ‒ | ‒ | ‒ | BS737_19 | full | 1 |  |
| Nirmala | f | 6 | ‒ | ‒ | ‒ | ‒ | ‒ | ‒ | BS739_19 | full | 1 |  |
| Fuxia | f | 3 | ‒ | ‒ | ‒ | ‒ | ‒ | ‒ | BT686_19 | full | 1 |  |
| Merula | f | 6 | ‒ | ‒ | ‒ | ‒ | ‒ | ‒ | BT691_19 | full | 1 |  |
| Humboldt | m | 5 | ‒ | ‒ | ‒ | ‒ | ‒ | ‒ | BT688_19 | full | 1 |  |
| Joanne | f | 5 | ‒ | ‒ | ‒ | ‒ | ‒ | ‒ | BT687_19 | full | 1 |  |
| Benita | f | 9 | ‒ | ‒ | ‒ | ‒ | ‒ | ‒ | BT690_19 | full | 1 |  |
| Laurita | f | 3 | ‒ | ‒ | ‒ | ‒ | ‒ | ‒ | BT694_19 | full | 1 |  |
| Adelie | f | 6 | ‒ | ‒ | ‒ | ‒ | ‒ | ‒ | BT693_19 | full | 1 |  |
| Selima | f | 6 | ‒ | ‒ | ‒ | ‒ | ‒ | ‒ | BS720_19 | full | 1 |  |
| Prosecco | f | 12 | ‒ | ‒ | ‒ | ‒ | ‒ | ‒ | BS572_19 | full | 1 |  |

**Table S2** Estimated mean longitude and latitude of wintering area(s) and stopover site(s), with the associated median arrival and departure dates, and the distance travelled using a “*prob.cutoff*” of 0.1, 0.2, 0.3, 0.4 and 0.5 for the “*stationary.migration.summary*” function for sixteen randomly selected full tracks of common terns deployed with a light-level geolocator in 2016, 2017, 2018 and/or 2019. Grey markings indicate unreliable estimations resulting in (i) a fragmentation (> two location estimations) of the wintering area(s) (within a range of 500 km) and/or the breeding area (within a range of 250 km), (ii) overlapping arrival and departure dates and (iii) no detection of the breeding area.

| **Z434_16** | **lon** | **lat** | **arrival** | **departure** | **dist** |
| --- | --- | --- | --- | --- | --- |
| **0.10** | 8.34 | 53.56 | NA | 11.09.2016 | 0 |
|  | -17.01 | 22.02 | 15.09.2016 | 15.03.2017 | 4105 |
|  | -16.39 | 24.51 | 23.03.2017 | 23.03.2017 | 283 |
|  | 8.32 | 53.47 | 10.04.2017 | NA | 3821 |
| **0.20** | 8.34 | 53.56 | NA | 11.09.2016 | 0 |
|  | -17.01 | 22.02 | 15.09.2016 | 15.03.2017 | 4105 |
|  | -15.22 | 26.31 | 31.03.2017 | 31.03.2017 | 509 |
|  | 8.32 | 53.47 | 10.04.2017 | NA | 3589 |
| **0.30** | 8.34 | 53.56 | NA | 11.09.2016 | 0 |
|  | -17.01 | 22.02 | 15.09.2016 | 15.03.2017 | 4105 |
|  | -15.22 | 26.31 | 31.03.2017 | 31.03.2017 | 509 |
|  | 8.29 | 53.39 | 10.04.2017 | NA | 3582 |
| **0.40** | 8.33 | 53.54 | NA | 11.09.2016 | 0 |
|  | -16.77 | 22.56 | 15.09.2016 | 30.03.2017 | 4038 |
|  | 8.29 | 53.39 | 10.04.2017 | NA | 4024 |
| **0.50** | 8.33 | 53.54 | NA | 11.09.2016 | 0 |
|  | -10.89 | 29.84 | 15.09.2016 | NA | 3058 |
|  |  |  |  |  |  |
| **Z440_16** | **lon** | **lat** | **arrival** | **departure** | **dist** |
| **0.10** | 8.33 | 53.55 | NA | 26.08.2016 | 0 |
|  | -16.52 | 10.93 | 02.09.2016 | 24.11.2016 | 5219 |
|  | -16.25 | 14.27 | 19.10.2016 | 01.02.2017 | 371 |
|  | -16.59 | 14.32 | 01.02.2017 | 01.02.2017 | 37 |
|  | -17.69 | 13.80 | 01.02.2017 | 26.02.2017 | 132 |
|  | -16.53 | 19.94 | 25.02.2017 | 25.02.2017 | 690 |
|  | -16.48 | 20.90 | 27.02.2017 | 06.04.2017 | 106 |
|  | -9.45 | 42.97 | 09.04.2017 | 23.04.2017 | 2534 |
|  | 8.28 | 53.56 | 25.04.2017 | NA | 1757 |
| **0.20** | 8.33 | 53.55 | NA | 26.08.2016 | 0 |
|  | -16.33 | 13.41 | 02.09.2016 | 01.02.2017 | 4955 |
|  | -16.62 | 14.30 | 02.02.2017 | 02.02.2017 | 103 |
|  | -17.16 | 16.47 | 24.01.2017 | 06.04.2017 | 248 |
|  | -16.48 | 20.90 | 27.02.2017 | 06.04.2017 | 495 |
|  | -9.45 | 42.97 | 09.04.2017 | 23.04.2017 | 2534 |
|  | 8.28 | 53.56 | 25.04.2017 | NA | 1758 |
| **0.30** | 8.33 | 53.55 | NA | 26.08.2016 | 0 |
|  | -16.35 | 13.47 | 02.09.2016 | 02.02.2017 | 4948 |
|  | -16.94 | 17.84 | 24.01.2017 | 06.04.2017 | 488 |
|  | -9.45 | 42.96 | 09.04.2017 | 23.04.2017 | 2874 |
|  | 8.28 | 53.56 | 25.04.2017 | NA | 1758 |
| **0.40** | 8.31 | 53.53 | NA | 26.08.2016 | 0 |
|  | -16.52 | 14.74 | 02.09.2016 | 06.04.2017 | 4821 |
|  | -9.45 | 42.97 | 09.04.2017 | 23.04.2017 | 3201 |
|  | 8.28 | 53.56 | 25.04.2017 | NA | 1758 |
| **0.50** | 8.31 | 53.53 | NA | 26.08.2016 | 0 |
|  | -16.52 | 14.75 | 02.09.2016 | 06.04.2017 | 4821 |
|  | -9.45 | 42.97 | 09.04.2017 | 23.04.2017 | 3200 |
|  | 8.11 | 53.56 | 25.04.2017 | NA | 1748 |
| **Z444_16** | **lon** | **lat** | **arrival** | **departure** | **dist** |
| **0.1** | 8.34 | 53.55 | NA | 29.08.2016 | 0 |
|  | 8.16 | 53.47 | 29.08.2016 | 29.08.2016 | 16 |
|  | -16.77 | 19.87 | 06.09.2016 | 01.04.2017 | 4296 |
|  | 7.97 | 53.35 | 09.04.2017 | NA | 4277 |
| **0.2** | 8.31 | 53.51 | NA | 29.08.2016 | 0 |
|  | -16.77 | 19.87 | 06.09.2016 | 01.04.2017 | 4306 |
|  | 7.97 | 53.35 | 09.04.2017 | NA | 4277 |
| **0.3** | 8.31 | 53.51 | NA | 29.08.2016 | 0 |
|  | -16.77 | 19.87 | 06.09.2016 | 01.04.2017 | 4306 |
|  | 7.97 | 53.35 | 09.04.2017 | NA | 4277 |
| **0.4** | 8.31 | 53.51 | NA | 29.08.2016 | 0 |
|  | -16.77 | 19.87 | 05.09.2016 | 01.04.2017 | 4306 |
|  | -11.16 | 39.45 | 01.04.2017 | 08.04.2017 | 2236 |
|  | 7.97 | 53.35 | 09.04.2017 | NA | 2120 |
| **0.5** | 8.31 | 53.51 | NA | 29.08.2016 | 0 |
|  | -16.77 | 19.87 | 05.09.2016 | 01.04.2017 | 4305 |
|  | -11.16 | 39.46 | 01.04.2017 | 08.04.2017 | 2237 |
|  | 7.97 | 53.35 | 09.04.2017 | NA | 2119 |
| **Z446_16** | **lon** | **lat** | **arrival** | **departure** | **dist** |
| **0.10** | 8.26 | 53.59 | NA | 30.08.2016 | 0 |
|  | -16.85 | 20.92 | 07.09.2016 | 19.11.2016 | 4208 |
|  | -16.79 | 20.47 | 19.11.2016 | 19.11.2016 | 50 |
|  | -16.46 | 19.19 | 28.10.2016 | 28.10.2016 | 146 |
|  | -16.28 | 18.36 | 19.11.2016 | 02.04.2017 | 94 |
|  | 8.33 | 53.54 | 11.04.2017 | NA | 4443 |
| **0.20** | 8.26 | 53.59 | NA | 30.08.2016 | 0 |
|  | -16.84 | 20.86 | 07.09.2016 | 19.11.2016 | 4214 |
|  | -16.46 | 19.19 | 28.10.2016 | 28.10.2016 | 189 |
|  | -16.28 | 18.36 | 19.11.2016 | 02.04.2017 | 94 |
|  | 8.33 | 53.54 | 11.04.2017 | NA | 4443 |
| **0.30** | 8.26 | 53.59 | NA | 30.08.2016 | 0 |
|  | -16.84 | 20.86 | 07.09.2016 | 19.11.2016 | 4214 |
|  | -16.16 | 18.86 | 19.11.2016 | 02.04.2017 | 232 |
|  | 8.33 | 53.54 | 11.04.2017 | NA | 4387 |
| **0.40** | 8.21 | 53.54 | NA | 30.08.2016 | 0 |
|  | -16.84 | 20.86 | 07.09.2016 | 19.11.2016 | 4208 |
|  | -16.14 | 18.94 | 19.11.2016 | 02.04.2017 | 224 |
|  | 8.09 | 53.08 | 10.04.2017 | NA | 4327 |
| **0.50** | 7.94 | 53.25 | NA | 30.08.2016 | 0 |
|  | -16.39 | 19.63 | 07.09.2016 | 02.04.2017 | 4276 |
|  | 8.09 | 53.08 | 10.04.2017 | NA | 4268 |
| **BD664_17** | **lon** | **lat** | **arrival** | **departure** | **dist** |
| **0.10** | 8.29 | 53.50 | NA | 28.08.2017 | 0 |
|  | -16.02 | 10.82 | 07.09.2017 | 04.02.2018 | 5206 |
|  | -13.76 | 8.77 | 05.02.2018 | 20.03.2018 | 336 |
|  | -18.28 | 17.00 | 20.03.2018 | 30.03.2018 | 1034 |
|  | -14.95 | 25.12 | 30.03.2018 | 09.04.2018 | 963 |
|  | 8.27 | 53.46 | 18.04.2018 | NA | 3693 |
| **0.20** | 8.29 | 53.50 | NA | 28.08.2017 | 0 |
|  | -16.01 | 10.91 | 06.09.2017 | 04.02.2018 | 5195 |
|  | -13.76 | 8.77 | 05.02.2018 | 20.03.2018 | 343 |
|  | -18.28 | 17.00 | 20.03.2018 | 30.03.2018 | 1034 |
|  | -15.52 | 24.44 | 28.03.2018 | 09.04.2018 | 873 |
|  | 8.27 | 53.46 | 18.04.2018 | NA | 3785 |
| **0.30** | 8.29 | 53.50 | NA | 28.08.2017 | 0 |
|  | -16.01 | 10.91 | 06.09.2017 | 04.02.2018 | 5196 |
|  | -13.76 | 8.77 | 05.02.2018 | 20.03.2018 | 342 |
|  | -16.59 | 21.16 | 20.03.2018 | 09.04.2018 | 1404 |
|  | 8.27 | 53.46 | 18.04.2018 | NA | 4161 |
| **0.40** | 8.29 | 53.50 | NA | 28.08.2017 | 0 |
|  | -16.01 | 10.91 | 06.09.2017 | 04.02.2018 | 5196 |
|  | -13.76 | 8.77 | 05.02.2018 | 20.03.2018 | 343 |
|  | -16.59 | 21.16 | 20.03.2018 | 09.04.2018 | 1405 |
|  | -11.51 | 29.57 | 09.04.2018 | 15.04.2018 | 1063 |
|  | 8.27 | 53.46 | 18.04.2018 | NA | 3101 |
| **0.50** | 8.29 | 53.50 | NA | 28.08.2017 | 0 |
|  | -16.01 | 10.91 | 06.09.2017 | 04.02.2018 | 5196 |
|  | -13.77 | 8.81 | 05.02.2018 | 20.03.2018 | 338 |
|  | -15.14 | 23.94 | 20.03.2018 | 16.04.2018 | 1681 |
|  | 8.18 | 53.35 | 18.04.2018 | NA | 3807 |
| **BD665_17** | **lon** | **lat** | **arrival** | **departure** | **dist** |
| **0.10** | 8.29 | 53.55 | NA | 18.09.2017 | 0 |
|  | 8.21 | 53.47 | 18.09.2017 | 18.09.2017 | 10 |
|  | -13.66 | 8.97 | 29.09.2017 | 30.03.2018 | 5311 |
|  | -16.30 | 19.10 | 30.03.2018 | 05.04.2018 | 1156 |
|  | 8.16 | 53.57 | 18.04.2018 | NA | 4363 |
| **0.20** | 8.27 | 53.51 | NA | 18.09.2017 | 0 |
|  | -13.66 | 8.98 | 29.09.2017 | 30.03.2018 | 5316 |
|  | -16.30 | 19.10 | 30.03.2018 | 05.04.2018 | 1155 |
|  | -15.20 | 27.36 | 05.04.2018 | 12.04.2018 | 921 |
|  | 8.16 | 53.57 | 18.04.2018 | NA | 3486 |
| **0.30** | 8.27 | 53.51 | NA | 18.09.2017 | 0 |
|  | -13.66 | 8.98 | 29.09.2017 | 30.03.2018 | 5316 |
|  | -16.30 | 19.11 | 30.03.2018 | 05.04.2018 | 1156 |
|  | -14.09 | 30.44 | 05.04.2018 | 17.04.2018 | 1275 |
|  | 8.16 | 53.57 | 18.04.2018 | NA | 3134 |
| **0.40** | 8.27 | 53.51 | NA | 18.09.2017 | 0 |
|  | -13.66 | 8.98 | 29.09.2017 | 30.03.2018 | 5316 |
|  | -16.30 | 19.10 | 30.03.2018 | 05.04.2018 | 1156 |
|  | -14.09 | 30.43 | 05.04.2018 | 17.04.2018 | 1275 |
|  | 8.16 | 53.57 | 18.04.2018 | NA | 3134 |
| **0.50** | 8.27 | 53.51 | NA | 18.09.2017 | 0 |
|  | -13.68 | 9.42 | 29.09.2017 | 30.03.2018 | 5270 |
|  | -16.30 | 19.10 | 30.03.2018 | 05.04.2018 | 1108 |
|  | -14.09 | 30.44 | 05.04.2018 | 17.04.2018 | 1276 |
|  | 8.16 | 53.57 | 18.04.2018 | NA | 3133 |
| **BD671_17** | **lon** | **lat** | **arrival** | **departure** | **dist** |
| **0.1** | 8.34 | 53.55 | NA | 22.07.2017 | 0 |
|  | 4.99 | 53.56 | 23.07.2017 | 30.08.2017 | 222 |
|  | -16.99 | 16.05 | 05.09.2017 | 16.09.2017 | 4581 |
|  | -1.94 | 7.33 | 17.09.2017 | 25.09.2017 | 1901 |
|  | 0.00 | 5.78 | 04.10.2017 | 23.02.2018 | 274 |
|  | -2.44 | 10.48 | 23.02.2018 | 04.03.2018 | 585 |
|  | -14.14 | 11.59 | 06.03.2018 | 22.03.2018 | 1284 |
|  | -16.96 | 18.90 | 22.03.2018 | 31.03.2018 | 864 |
|  | -0.60 | 43.39 | 08.04.2018 | 14.04.2018 | 3118 |
|  | 8.28 | 53.50 | 15.04.2018 | NA | 1300 |
| **0.2** | 8.34 | 53.55 | NA | 22.07.2017 | 0 |
|  | 4.96 | 53.52 | 23.07.2017 | 30.08.2017 | 224 |
|  | -16.97 | 16.16 | 05.09.2017 | 16.09.2017 | 4564 |
|  | -2.01 | 7.33 | 17.09.2017 | 26.09.2017 | 1899 |
|  | -0.15 | 6.07 | 26.09.2017 | 01.03.2018 | 248 |
|  | -14.12 | 11.57 | 06.03.2018 | 22.03.2018 | 1652 |
|  | -16.88 | 19.17 | 22.03.2018 | 01.04.2018 | 891 |
|  | -0.60 | 43.39 | 08.04.2018 | 14.04.2018 | 3088 |
|  | 8.26 | 53.46 | 15.04.2018 | NA | 1296 |
| **0.3** | 8.39 | 53.51 | NA | 22.07.2017 | 0 |
|  | 4.96 | 53.52 | 23.07.2017 | 30.08.2017 | 228 |
|  | -16.97 | 16.16 | 05.09.2017 | 16.09.2017 | 4563 |
|  | -2.00 | 7.28 | 17.09.2017 | 17.09.2017 | 1903 |
|  | -0.15 | 6.08 | 26.09.2017 | 01.03.2018 | 243 |
|  | -14.12 | 11.57 | 06.03.2018 | 22.03.2018 | 1652 |
|  | -16.88 | 19.17 | 22.03.2018 | 01.04.2018 | 891 |
|  | -0.60 | 43.39 | 08.04.2018 | 14.04.2018 | 3088 |
|  | 8.26 | 53.46 | 15.04.2018 | NA | 1296 |
| **0.4** | 8.39 | 53.51 | NA | 22.07.2017 | 0 |
|  | 4.79 | 53.21 | 23.07.2017 | 30.08.2017 | 242 |
|  | -17.03 | 17.63 | 04.09.2017 | 16.09.2017 | 4379 |
|  | -2.00 | 7.29 | 17.09.2017 | 17.09.2017 | 1992 |
|  | -0.15 | 6.08 | 26.09.2017 | 01.03.2018 | 244 |
|  | -15.09 | 14.33 | 06.03.2018 | 01.04.2018 | 1873 |
|  | -0.58 | 43.79 | 08.04.2018 | 15.04.2018 | 3545 |
|  | 8.17 | 53.36 | 15.04.2018 | NA | 1243 |
| **0.5** | 8.39 | 53.51 | NA | 22.07.2017 | 0 |
|  | 4.79 | 53.21 | 23.07.2017 | 30.08.2017 | 242 |
|  | -17.03 | 17.63 | 04.09.2017 | 16.09.2017 | 4379 |
|  | -0.25 | 6.14 | 22.09.2017 | 04.03.2018 | 2224 |
|  | -14.76 | 14.21 | 05.03.2018 | 01.04.2018 | 1823 |
|  | 6.09 | 51.08 | 08.04.2018 | NA | 4499 |
| **Z435_17** | **lon** | **lat** | **arrival** | **departure** | **dist** |
| **0.10** | 8.34 | 53.55 | NA | 31.08.2017 | 0 |
|  | -16.23 | 12.35 | 08.09.2017 | 04.04.2018 | 5060 |
|  | 8.27 | 53.21 | 18.04.2018 | NA | 5028 |
| **0.20** | 8.33 | 53.53 | NA | 31.08.2017 | 0 |
|  | -16.24 | 12.36 | 08.09.2017 | 04.04.2018 | 5058 |
|  | -15.93 | 22.59 | 04.04.2018 | 10.04.2018 | 1133 |
|  | 8.27 | 53.21 | 18.04.2018 | NA | 3968 |
| **0.30** | 8.32 | 53.50 | NA | 31.08.2017 | 0 |
|  | -16.25 | 12.43 | 07.09.2017 | 04.04.2018 | 5048 |
|  | -15.93 | 22.59 | 04.04.2018 | 10.04.2018 | 1126 |
|  | 8.28 | 53.21 | 18.04.2018 | NA | 3968 |
| **0.40** | 8.32 | 53.50 | NA | 31.08.2017 | 0 |
|  | -16.24 | 12.69 | 07.09.2017 | 10.04.2018 | 5021 |
|  | 8.28 | 53.21 | 18.04.2018 | NA | 4993 |
| **0.50** | 8.32 | 53.50 | NA | 31.08.2017 | 0 |
|  | -16.24 | 12.68 | 07.09.2017 | 10.04.2018 | 5021 |
|  | 8.28 | 53.21 | 18.04.2018 | NA | 4993 |
| **BD277_18** | **lon** | **lat** | **arrival** | **departure** | **dist** |
| **0.10** | 8.34 | 53.55 | NA | 04.09.2018 | 0 |
|  | -16.87 | 14.49 | 09.09.2018 | 14.04.2019 | 4863 |
|  | -17.24 | 14.75 | 28.01.2019 | 14.04.2019 | 49 |
|  | 8.09 | 53.47 | 24.04.2019 | NA | 4835 |
| **0.20** | 8.34 | 53.55 | NA | 04.09.2018 | 0 |
|  | -17.00 | 14.60 | 09.09.2018 | 14.04.2019 | 4857 |
|  | 8.09 | 53.47 | 24.04.2019 | NA | 4840 |
| **0.30** | 8.32 | 53.50 | NA | 04.09.2018 | 0 |
|  | -17.00 | 14.60 | 09.09.2018 | 14.04.2019 | 4853 |
|  | 8.09 | 53.47 | 24.04.2019 | NA | 4840 |
| **0.40** | 8.32 | 53.51 | NA | 04.09.2018 | 0 |
|  | -17.00 | 14.60 | 09.09.2018 | 14.04.2019 | 4853 |
|  | -10.52 | 39.01 | 17.04.2019 | 23.04.2019 | 2778 |
|  | 8.09 | 53.47 | 24.04.2019 | NA | 2141 |
| **0.50** | 8.32 | 53.51 | NA | 04.09.2018 | 0 |
|  | -17.00 | 14.74 | 09.09.2018 | 14.04.2019 | 4838 |
|  | -10.52 | 39.01 | 17.04.2019 | 23.04.2019 | 2763 |
|  | 8.09 | 53.47 | 24.04.2019 | NA | 2141 |
| **BD672_18** | **lon** | **lat** | **arrival** | **departure** | **dist** |
| **0.10** | 8.34 | 53.55 | NA | 30.08.2018 | 0 |
|  | -16.58 | 18.12 | 04.09.2018 | 04.09.2018 | 4480 |
|  | -17.52 | 8.98 | 17.09.2018 | 06.10.2018 | 1017 |
|  | -16.53 | 18.55 | 06.10.2018 | 27.03.2019 | 1065 |
|  | -11.40 | 36.64 | 27.03.2019 | 06.04.2019 | 2067 |
|  | 7.48 | 53.16 | 08.04.2019 | NA | 2350 |
| **0.20** | 8.34 | 53.55 | NA | 30.08.2018 | 0 |
|  | -16.62 | 17.70 | 04.09.2018 | 27.03.2019 | 4525 |
|  | -11.40 | 36.64 | 27.03.2019 | 06.04.2019 | 2161 |
|  | 7.45 | 53.14 | 08.04.2019 | NA | 2347 |
| **0.30** | 8.331 | 53.52 | NA | 30.08.2018 | 0 |
|  | -16.6 | 17.70 | 04.09.2018 | 27.03.2019 | 4522 |
|  | -11.4 | 36.64 | 27.03.2019 | 06.04.2019 | 2161 |
|  | 7.454 | 53.14 | 08.04.2019 | NA | 2347 |
| **0.40** | 8.33 | 53.52 | NA | 30.08.2018 | 0 |
|  | -16.62 | 17.70 | 04.09.2018 | 27.03.2019 | 4522 |
|  | -11.23 | 37.17 | 27.03.2019 | 07.04.2019 | 2222 |
|  | 7.45 | 53.15 | 08.04.2019 | NA | 2290 |
| **0.50** | 8.33 | 53.52 | NA | 30.08.2018 | 0 |
|  | -16.62 | 17.70 | 04.09.2018 | 27.03.2019 | 4522 |
|  | -11.23 | 37.17 | 27.03.2019 | 07.04.2019 | 2221 |
|  | 7.45 | 53.14 | 08.04.2019 | NA | 2290 |
| **BL164_18** | **lon** | **lat** | **arrival** | **departure** | **dist** |
| **0.10** | 8.33 | 53.54 | NA | 17.09.2018 | 0 |
|  | 2.13 | 47.51 | 17.09.2018 | 23.09.2018 | 801 |
|  | -16.69 | 19.84 | 26.09.2018 | 27.12.2018 | 3509 |
|  | -17.04 | 15.68 | 28.12.2018 | 09.02.2019 | 462 |
|  | -17.14 | 21.32 | 09.02.2019 | 16.03.2019 | 624 |
|  | -13.76 | 28.03 | 16.03.2019 | 15.04.2019 | 818 |
|  | 8.06 | 53.61 | 20.04.2019 | NA | 3355 |
| **0.20** | 8.33 | 53.53 | NA | 17.09.2018 | 0 |
|  | 2.13 | 47.51 | 17.09.2018 | 23.09.2018 | 800 |
|  | -16.69 | 19.85 | 26.09.2018 | 27.12.2018 | 3509 |
|  | -17.04 | 15.68 | 28.12.2018 | 09.02.2019 | 462 |
|  | -17.14 | 21.11 | 16.03.2019 | 16.03.2019 | 601 |
|  | -13.76 | 28.03 | 16.03.2019 | 15.04.2019 | 839 |
|  | 8.06 | 53.61 | 20.04.2019 | NA | 3355 |
| **0.30** | 8.33 | 53.53 | NA | 17.09.2018 | 0 |
|  | 2.13 | 47.51 | 17.09.2018 | 23.09.2018 | 800 |
|  | -16.69 | 19.83 | 26.09.2018 | 27.12.2018 | 3511 |
|  | -17.08 | 18.22 | 28.12.2018 | 16.03.2019 | 183 |
|  | -13.76 | 28.03 | 16.03.2019 | 15.04.2019 | 1138 |
|  | 8.06 | 53.61 | 20.04.2019 | NA | 3355 |
| **0.40** | 8.33 | 53.53 | NA | 17.09.2018 | 0 |
|  | 2.13 | 47.51 | 17.09.2018 | 23.09.2018 | 800 |
|  | -16.69 | 19.82 | 26.09.2018 | 27.12.2018 | 3512 |
|  | -17.08 | 18.22 | 28.12.2018 | 16.03.2019 | 182 |
|  | -13.76 | 28.03 | 16.03.2019 | 15.04.2019 | 1138 |
|  | 8.06 | 53.61 | 20.04.2019 | NA | 3355 |
| **0.50** | 8.33 | 53.53 | NA | 17.09.2018 | 0 |
|  | 2.13 | 47.51 | 17.09.2018 | 23.09.2018 | 800 |
|  | -16.69 | 19.82 | 26.09.2018 | 27.12.2018 | 3512 |
|  | -17.09 | 18.22 | 28.12.2018 | 16.03.2019 | 182 |
|  | -13.73 | 28.10 | 16.03.2019 | 15.04.2019 | 1147 |
|  | 8.06 | 53.61 | 20.04.2019 | NA | 3347 |
| **BL126_19** | **lon** | **lat** | **arrival** | **departure** | **dist** |
| **0.10** | 8.33 | 53.55 | NA | 25.08.2019 | 0 |
|  | 7.48 | 53.32 | 25.08.2019 | 10.09.2019 | 62 |
|  | 5.69 | 53.88 | 25.08.2019 | 01.10.2019 | 134 |
|  | 4.50 | 53.87 | 01.10.2019 | 01.10.2019 | 78 |
|  | -16.66 | 19.03 | 09.10.2019 | 04.04.2020 | 4270 |
|  | -4.83 | 43.87 | 12.04.2020 | 18.04.2020 | 2967 |
|  | 8.03 | 53.60 | 20.04.2020 | NA | 1433 |
| **0.20** | 8.03 | 53.46 | NA | 25.08.2019 | 0 |
|  | 5.69 | 53.88 | 25.08.2019 | 01.10.2019 | 162 |
|  | 4.50 | 53.87 | 01.10.2019 | 01.10.2019 | 78 |
|  | -16.66 | 19.08 | 09.10.2019 | 04.04.2020 | 4265 |
|  | -4.83 | 43.87 | 12.04.2020 | 18.04.2020 | 2962 |
|  | 8.03 | 53.60 | 20.04.2020 | NA | 1433 |
| **0.30** | 8.03 | 53.46 | NA | 25.08.2019 | 0 |
|  | 5.69 | 53.88 | 25.08.2019 | 01.10.2019 | 162 |
|  | 4.50 | 53.88 | 01.10.2019 | 01.10.2019 | 78 |
|  | -16.66 | 19.14 | 09.10.2019 | 04.04.2020 | 4260 |
|  | -4.71 | 43.96 | 12.04.2020 | 19.04.2020 | 2969 |
|  | 8.03 | 53.60 | 20.04.2020 | NA | 1419 |
| **0.40** | 7.41 | 53.54 | NA | 01.10.2019 | 0 |
|  | -16.65 | 19.16 | 09.10.2019 | 04.04.2020 | 4338 |
|  | -9.66 | 37.99 | 07.04.2020 | 13.04.2020 | 2194 |
|  | -4.71 | 43.95 | 12.04.2020 | 19.04.2020 | 782 |
|  | 8.03 | 53.60 | 20.04.2020 | NA | 1420 |
| **0.50** | 7.41 | 53.54 | NA | 01.10.2019 | 0 |
|  | -16.65 | 19.16 | 09.10.2019 | 04.04.2020 | 4338 |
|  | -10.40 | 35.36 | 04.04.2020 | 13.04.2020 | 1897 |
|  | -4.71 | 43.95 | 12.04.2020 | 19.04.2020 | 1071 |
|  | 8.03 | 53.60 | 20.04.2020 | NA | 1420 |
| **BS721_19** | **lon** | **lat** | **arrival** | **departure** | **dist** |
| **0.10** | 8.34 | 53.55 | NA | 27.08.2019 | 0 |
|  | -16.57 | 18.23 | 05.09.2019 | 31.03.2020 | 4469 |
|  | -16.68 | 16.69 | 31.03.2020 | 31.03.2020 | 170 |
|  | -16.62 | 19.75 | 31.03.2020 | 31.03.2020 | 339 |
|  | 7.64 | 53.93 | 10.04.2020 | NA | 4320 |
| **0.20** | 8.34 | 53.55 | NA | 27.08.2019 | 0 |
|  | -16.58 | 18.29 | 01.09.2019 | 31.03.2020 | 4463 |
|  | 7.64 | 53.93 | 10.04.2020 | NA | 4469 |
| **0.30** | 8.34 | 53.55 | NA | 27.08.2019 | 0 |
|  | -16.58 | 18.29 | 01.09.2019 | 31.03.2020 | 4463 |
|  | 7.59 | 53.88 | 10.04.2020 | NA | 4462 |
| **0.40** | 8.34 | 53.55 | NA | 27.08.2019 | 0 |
|  | -16.58 | 18.29 | 01.09.2019 | 31.03.2020 | 4463 |
|  | 7.59 | 53.88 | 10.04.2020 | NA | 4461 |
| **0.50** | 8.34 | 53.55 | NA | 27.08.2019 | 0 |
|  | -16.58 | 18.29 | 01.09.2019 | 31.03.2020 | 4463 |
|  | -6.66 | 46.88 | 04.04.2020 | 10.04.2020 | 3299 |
|  | 7.58 | 53.88 | 10.04.2020 | NA | 1273 |
| **BS724_19** | **lon** | **lat** | **arrival** | **departure** | **dist** |
| **0.10** | 8.33 | 53.54 | NA | 11.09.2019 | 0 |
|  | -16.77 | 20.08 | 17.09.2019 | 17.09.2019 | 4287 |
|  | -16.33 | 10.47 | 01.10.2019 | 22.03.2020 | 1065 |
|  | -16.27 | 13.31 | 27.03.2020 | 27.03.2020 | 315 |
|  | -16.26 | 15.67 | 02.04.2020 | 02.04.2020 | 261 |
|  | -8.26 | 47.34 | 07.04.2020 | 16.04.2020 | 3589 |
|  | 8.01 | 53.57 | 20.04.2020 | NA | 1343 |
| **0.20** | 8.33 | 53.54 | NA | 11.09.2019 | 0 |
|  | -16.36 | 11.22 | 24.09.2019 | 22.03.2020 | 5181 |
|  | -16.30 | 15.58 | 03.04.2020 | 03.04.2020 | 482 |
|  | -8.26 | 47.34 | 07.04.2020 | 16.04.2020 | 3600 |
|  | 8.00 | 53.58 | 20.04.2020 | NA | 1342 |
| **0.30** | 8.33 | 53.54 | NA | 11.09.2019 | 0 |
|  | -16.36 | 11.22 | 24.09.2019 | 22.03.2020 | 5181 |
|  | -16.30 | 15.57 | 03.04.2020 | 03.04.2020 | 481 |
|  | -8.90 | 46.30 | 05.04.2020 | 16.04.2020 | 3476 |
|  | 7.99 | 53.58 | 20.04.2020 | NA | 1453 |
| **0.40** | 8.33 | 53.54 | NA | 11.09.2019 | 0 |
|  | -16.33 | 11.88 | 19.09.2019 | 02.04.2020 | 5111 |
|  | -8.90 | 46.30 | 05.04.2020 | 16.04.2020 | 3879 |
|  | 7.99 | 53.58 | 20.04.2020 | NA | 1452 |
| **0.50** | 8.33 | 53.54 | NA | 11.09.2019 | 0 |
|  | -16.01 | 13.35 | 17.09.2019 | 16.04.2020 | 4972 |
|  | 7.99 | 53.58 | 20.04.2020 | NA | 4963 |
| **BS729_19** | **lon** | **lat** | **arrival** | **departure** | **dist** |
| **0.10** | 8.33 | 53.54 | NA | 17.08.2019 | 0 |
|  | 8.16 | 53.28 | 01.10.2019 | 01.10.2019 | 31 |
|  | -16.91 | 19.54 | 06.10.2019 | 06.10.2019 | 4319 |
|  | -16.63 | 16.92 | 06.10.2019 | 05.04.2020 | 292 |
|  | -16.65 | 15.70 | 05.04.2020 | 05.04.2020 | 135 |
|  | -17.47 | 27.45 | 12.04.2020 | 12.04.2020 | 1304 |
|  | 7.98 | 53.77 | 19.04.2020 | NA | 3589 |
| **0.20** | 8.23 | 53.40 | NA | 01.10.2019 | 0 |
|  | -16.65 | 17.03 | 06.10.2019 | 05.04.2020 | 4577 |
|  | -17.47 | 27.46 | 12.04.2020 | 12.04.2020 | 1158 |
|  | 7.98 | 53.77 | 19.04.2020 | NA | 3588 |
| **0.30** | 8.23 | 53.40 | NA | 01.10.2019 | 0 |
|  | -16.65 | 17.03 | 06.10.2019 | 05.04.2020 | 4577 |
|  | -17.45 | 27.23 | 12.04.2020 | 12.04.2020 | 1132 |
|  | 7.98 | 53.77 | 19.04.2020 | NA | 3610 |
| **0.40** | 8.23 | 53.40 | NA | 01.10.2019 | 0 |
|  | -16.68 | 17.38 | 06.10.2019 | 06.04.2020 | 4542 |
|  | 7.92 | 53.74 | 19.04.2020 | NA | 4560 |
| **0.50** | 8.23 | 53.40 | NA | 01.10.2019 | 0 |
|  | -16.67 | 17.43 | 06.10.2019 | 06.04.2020 | 4537 |
|  | -8.85 | 46.33 | 14.04.2020 | 19.04.2020 | 3286 |
|  | 7.92 | 53.74 | 19.04.2020 | NA | 1451 |
| **BU573_19** | **lon** | **lat** | **arrival** | **departure** | **dist** |
| **0.10** | 8.27 | 53.43 | NA | 30.09.2019 | 0 |
|  | -16.47 | 16.89 | 10.10.2019 | 06.11.2019 | 4589 |
|  | -16.48 | 10.78 | 15.11.2019 | 15.04.2020 | 676 |
|  | 7.94 | 53.96 | 28.04.2020 | NA | 5256 |
| **0.20** | 8.27 | 53.43 | NA | 30.09.2019 | 0 |
|  | -4.72 | 36.89 | 03.10.2019 | 09.10.2019 | 2096 |
|  | -16.46 | 16.92 | 10.10.2019 | 06.11.2019 | 2495 |
|  | -16.48 | 10.79 | 15.11.2019 | 15.04.2020 | 678 |
|  | 7.94 | 53.96 | 28.04.2020 | NA | 5255 |
| **0.30** | 8.27 | 53.43 | NA | 30.09.2019 | 0 |
|  | -4.72 | 36.88 | 03.10.2019 | 09.10.2019 | 2096 |
|  | -16.46 | 16.92 | 10.10.2019 | 06.11.2019 | 2495 |
|  | -16.48 | 10.84 | 15.11.2019 | 15.04.2020 | 673 |
|  | 7.94 | 53.96 | 28.04.2020 | NA | 5250 |
| **0.40** | 8.27 | 53.43 | NA | 30.09.2019 | 0 |
|  | -4.72 | 36.88 | 03.10.2019 | 09.10.2019 | 2096 |
|  | -16.46 | 16.92 | 10.10.2019 | 06.11.2019 | 2496 |
|  | -16.48 | 10.84 | 15.11.2019 | 15.04.2020 | 673 |
|  | 7.93 | 53.96 | 28.04.2020 | NA | 5250 |
| **0.50** | 8.24 | 53.24 | NA | 01.10.2019 | 0 |
|  | -4.72 | 36.89 | 03.10.2019 | 09.10.2019 | 2077 |
|  | -16.48 | 11.81 | 10.10.2019 | 15.04.2020 | 3017 |
|  | 7.94 | 53.96 | 28.04.2020 | NA | 5148 |

**Table S3** Track estimations with a “*prob.cutoff*” of 0.1, 0.2, 0.3, 0.4 and 0.5 for the sixteen randomly selected full tracks of common terns deployed a with light-level geolocator in 2016, 2017, 2018 and/or 2019 (Table S2), classified as reliable (YES) or unreliable (x).

|  |  | **prob.cutoff** | | | | |
| --- | --- | --- | --- | --- | --- | --- |
| **geolocator** | **year** | **0.1** | **0.2** | **0.3** | **0.4** | **0.5** |
| Z434_16 | 2016 | x | x | x | **YES** | x |
| Z440_16 | 2016 | x | x | YES | **YES** | YES |
| Z444_16 | 2016 | x | YES | YES | **YES** | YES |
| Z446_16 | 2016 | x | x | YES | **YES** | YES |
| BD664_17 | 2017 | YES | YES | YES | **YES** | YES |
| BD665_17 | 2017 | x | YES | YES | **YES** | YES |
| BD671_17 | 2017 | YES | YES | YES | **YES** | YES |
| Z435_17 | 2017 | YES | YES | YES | **YES** | YES |
| BD277_18 | 2018 | YES | YES | YES | **YES** | YES |
| BD672_18 | 2018 | x | YES | YES | **YES** | YES |
| BL164_18 | 2018 | YES | x | YES | **YES** | YES |
| BL126_19 | 2019 | x | x | x | **(YES)*** | (YES)* |
| BS721_19 | 2019 | x | YES | YES | **YES** | YES |
| BS724_19 | 2019 | x | x | YES | **YES** | YES |
| BS729_19 | 2019 | x | x | x | **YES** | YES |
| BU573_19 | 2019 | YES | YES | YES | **YES** | YES |
| **percentage of tracks with reliable estimations (%)** | | 38 | 56 | 82 | **100** | 94 |

*Note: departure date from one stopover site and arrival date at the next stopover site overlapped, which indicates an uncertainty of arrival and departure date estimations of stopover site(s) by the “*stationary.migration.summary*” function.

**Table S4** Mean repeatability (R) with standard error (SE), 95% confidence intervals (CI) and the p-values of the longitude and latitude of wintering area using all tracks (A) and when excluding partially incomplete tracks (i.e. tracks with data for less than two months in the wintering area) (B).

|  | **A. repeatability including all tracks** | | | **B. repeatability excluding non-full tracks** | | |
| --- | --- | --- | --- | --- | --- | --- |
| **trait** | R ± SE | CI | p-value | R ± SE | CI | p-value |
| wintering area  longitude | 0.998 ± 0.001 | 0.997‒0.999 | **<0.001** | 0.998 ± 0.001 | 0.996‒0.999 | **<0.001** |
| wintering area  latitude | 0.979 ± 0.005 | 0.970‒0.988 | **<0.001** | 0.978 ± 0.006 | 0.967‒0.988 | **<0.001** |

**Table S5** Mean repeatability (R) with standard error (SE), 95% confidence intervals (CI) and the p-values of stopover probability during autumn and spring migration based on the Link-scale and Original-scale approximation.

|  | **repeatability of stopover probability** | | | | | |
| --- | --- | --- | --- | --- | --- | --- |
| **trait** | Link-scale R ± SE | CI | p-value | Original-scale R ± SE | CI | p-value |
| autumn migration | 0.982 ± 0.008 | 0.983‒0.998 | **<0.001** | 0.413 ± 12.818 | 0.327‒0.658 | **<0.001** |
| spring migration | 0.269 ± 0.221 | 0.000‒0.963 | **0.031** | 0.319 ± 6.755 | 0.000‒26.028 | **0.031** |

**Table S6** Estimated mean longitude and latitude of stopover site(s) provided by the “*stationary.migration.summary*” function and the sum of stopover site(s) during autumn and spring migration for each common tern deployed with a light-level geolocator in 2016, 2017, 2018 and/or 2019. In cases of geolocator failure, “x” indicates the lack of available data.

|  |  |  |  | **autumn migration** | | | | | **spring migration** | | | | | | | |  |
| --- | --- | --- | --- | --- | --- | --- | --- | --- | --- | --- | --- | --- | --- | --- | --- | --- | --- |
| **individual** | **year** | **geo** | **sex** | **lon_1** | **lat_1** | **lon_2** | **lat_2** | **sum** | **lon_1** | **lat_1** | **lon_2** | **lat_2** | **lon_3** | **lat_3** | **lon_4** | **lat_4** | **sum** |
| Abel | 2016 | Z444_16 | m | ‒ | ‒ | ‒ | ‒ | 0 | -11.16 | 39.46 | ‒ | ‒ | ‒ | ‒ | ‒ | ‒ | 1 |
| Abel | 2017 | BD666_17 | m | ‒ | ‒ | ‒ | ‒ | 0 | ‒ | ‒ | ‒ | ‒ | ‒ | ‒ | ‒ | ‒ | 0 |
| Abel | 2018 | BL127_18 | m | ‒ | ‒ | ‒ | ‒ | 0 | x | x | x | x | x | x | x | x | x |
| Adelie | 2019 | BT693_19 | f | ‒ | ‒ | ‒ | ‒ | 0 | -16.73 | 23.88 | -14.60 | 30.88 | -7.94 | 46.94 | ‒ | ‒ | 3 |
| Anita | 2016 | Z450_16 | f | ‒ | ‒ | ‒ | ‒ | 0 | ‒ | ‒ | ‒ | ‒ | ‒ | ‒ | ‒ | ‒ | 0 |
| Anita | 2017 | BD670_17 | f | ‒ | ‒ | ‒ | ‒ | 0 | ‒ | ‒ | ‒ | ‒ | ‒ | ‒ | ‒ | ‒ | 0 |
| Anita | 2018 | BL161_18 | f | -9.72 | 40.33 | ‒ | ‒ | 1 | x | x | x | x | x | x | x | x | x |
| Antoine | 2018 | BD668_18 | m | ‒ | ‒ | ‒ | ‒ | 0 | -12.62 | 28.30 | -7.09 | 42.76 | ‒ | ‒ | ‒ | ‒ | 2 |
| Antoine | 2019 | BS731_19 | m | ‒ | ‒ | ‒ | ‒ | 0 | -15.98 | 9.29 | -17.58 | 19.26 | -1.82 | 46.83 | ‒ | ‒ | 3 |
| Aristide | 2018 | BL155_18 | m | ‒ | ‒ | ‒ | ‒ | 0 | x | x | x | x | x | x | x | x | x |
| Armando | 2016 | Z447_16 | m | ‒ | ‒ | ‒ | ‒ | 0 | ‒ | ‒ | ‒ | ‒ | ‒ | ‒ | ‒ | ‒ | 0 |
| Armando | 2017 | BD276_17 | m | ‒ | ‒ | ‒ | ‒ | 0 | -10.68 | 31.17 | ‒ | ‒ | ‒ | ‒ | ‒ | ‒ | 1 |
| Armando | 2018 | BL156_18 | m | ‒ | ‒ | ‒ | ‒ | 0 | x | x | x | x | x | x | x | x | x |
| Armando | 2019 | BS733_19 | m | -10.05 | 41.53 | ‒ | ‒ | 1 | -10.46 | 39.07 | ‒ | ‒ | ‒ | ‒ | ‒ | ‒ | 1 |
| Arno | 2017 | Z434_17 | m | ‒ | ‒ | ‒ | ‒ | 0 | ‒ | ‒ | ‒ | ‒ | ‒ | ‒ | ‒ | ‒ | 0 |
| Auguste | 2016 | Z441_16 | f | ‒ | ‒ | ‒ | ‒ | 0 | x | x | x | x | x | x | x | x | x |
| Autumn | 2019 | BS738_19 | f | -15.26 | 23.64 | ‒ | ‒ | 1 | -4.08 | -1.29 | -14.18 | 7.93 | -16.78 | 18.95 | -15.24 | 30.85 | 4 |
| Bea | 2016 | Z452_16 | f | ‒ | ‒ | ‒ | ‒ | 0 | -10.79 | 39.28 | -6.79 | 45.85 | ‒ | ‒ | ‒ | ‒ | 2 |
| Bea | 2017 | BD675_17 | f | ‒ | ‒ | ‒ | ‒ | 0 | x | x | x | x | x | x | x | x | x |
| Beccy | 2018 | BL141_18 | f | ‒ | ‒ | ‒ | ‒ | 0 | ‒ | ‒ | ‒ | ‒ | ‒ | ‒ | ‒ | ‒ | 0 |
| Beccy | 2019 | BS702_19 | f | ‒ | ‒ | ‒ | ‒ | 0 | ‒ | ‒ | ‒ | ‒ | ‒ | ‒ | ‒ | ‒ | 0 |
| Benita | 2019 | BT690_19 | f | -1.64 | 46.06 | ‒ | ‒ | 1 | -10.41 | 36.85 | ‒ | ‒ | ‒ | ‒ | ‒ | ‒ | 1 |
| Bibo | 2016 | Z594_16 | m | ‒ | ‒ | ‒ | ‒ | 0 | ‒ | ‒ | ‒ | ‒ | ‒ | ‒ | ‒ | ‒ | 0 |
| Bibo | 2017 | BD672_17 | m | -0.13 | 48.32 | ‒ | ‒ | 1 | ‒ | ‒ | ‒ | ‒ | ‒ | ‒ | ‒ | ‒ | 0 |
| Bibo | 2018 | BL132_18 | m | -16.51 | 24.62 | ‒ | ‒ | 1 | x | x | x | x | x | x | x | x | x |
| Bibo | 2019 | BS715_19 | m | -1.28 | 45.67 | ‒ | ‒ | 1 | ‒ | ‒ | ‒ | ‒ | ‒ | ‒ | ‒ | ‒ | 0 |
| Blake | 2017 | Z446_17 | m | ‒ | ‒ | ‒ | ‒ | 0 | ‒ | ‒ | ‒ | ‒ | ‒ | ‒ | ‒ | ‒ | 0 |
| Blake | 2018 | BL145_18 | m | ‒ | ‒ | ‒ | ‒ | 0 | x | x | x | x | x | x | x | x | x |
| Blake | 2019 | BS714_19 | m | 0.22 | 52.47 | ‒ | ‒ | 1 | ‒ | ‒ | ‒ | ‒ | ‒ | ‒ | ‒ | ‒ | 0 |
| Bofur | 2019 | BS724_19 | m | ‒ | ‒ | ‒ | ‒ | 0 | -8.90 | 46.30 | ‒ | ‒ | ‒ | ‒ | ‒ | ‒ | 1 |
| Cosima | 2016 | Z443_16 | f | -16.31 | 18.76 | ‒ | ‒ | 1 | -17.25 | 24.92 | ‒ | ‒ | ‒ | ‒ | ‒ | ‒ | 1 |
| Cosima | 2017 | BD680_17 | f | -17.05 | 18.84 | ‒ | ‒ | 1 | -18.63 | 12.70 | -15.05 | 24.85 | ‒ | ‒ | ‒ | ‒ | 2 |
| Cosima | 2018 | BL158_18 | f | -17.25 | 21.12 | ‒ | ‒ | 1 | x | x | x | x | x | x | x | x | x |
| Cosima | 2019 | BS708_19 | f | -16.66 | 21.08 | ‒ | ‒ | 1 | x | x | x | x | x | x | x | x | x |
| Cupido | 2019 | BS735_19 | m | -16.86 | 18.60 | ‒ | ‒ | 1 | -16.25 | 20.36 | ‒ | ‒ | ‒ | ‒ | ‒ | ‒ | 1 |
| Danilo | 2016 | Z449_16 | m | -16.39 | 27.51 | ‒ | ‒ | 1 | -15.75 | 21.69 | ‒ | ‒ | ‒ | ‒ | ‒ | ‒ | 1 |
| Danilo | 2017 | BD668_17 | m | -17.38 | 14.81 | ‒ | ‒ | 1 | -17.12 | 18.06 | ‒ | ‒ | ‒ | ‒ | ‒ | ‒ | 1 |
| Danilo | 2018 | BL153_18 | m | ‒ | ‒ | ‒ | ‒ | 0 | x | x | x | x | x | x | x | x | x |
| Djamila | 2016 | Z448_16 | f | -12.24 | 30.58 | ‒ | ‒ | 1 | -16.64 | 15.26 | ‒ | ‒ | ‒ | ‒ | ‒ | ‒ | 1 |
| Djamila | 2017 | BD673_17 | f | -16.57 | 18.91 | -16.93 | 21.86 | 2 | -16.22 | 21.66 | ‒ | ‒ | ‒ | ‒ | ‒ | ‒ | 1 |
| Djamila | 2018 | BD679_18 | f | ‒ | ‒ | ‒ | ‒ | 0 | -16.63 | 20.64 | ‒ | ‒ | ‒ | ‒ | ‒ | ‒ | 1 |
| Djamila | 2019 | BS728_19 | f | -16.42 | 21.24 | ‒ | ‒ | 1 | -17.34 | 19.90 | ‒ | ‒ | ‒ | ‒ | ‒ | ‒ | 1 |
| Elvira | 2016 | Z436_16 | f | ‒ | ‒ | ‒ | ‒ | 0 | -14.64 | 16.82 | -9.39 | 45.03 | ‒ | ‒ | ‒ | ‒ | 2 |
| Elvira | 2017 | BD664_17 | f | ‒ | ‒ | ‒ | ‒ | 0 | -16.59 | 21.16 | -11.51 | 29.58 | ‒ | ‒ | ‒ | ‒ | 2 |
| Elvira | 2018 | BL133_18 | f | ‒ | ‒ | ‒ | ‒ | 0 | x | x | x | x | x | x | x | x | x |
| Elvira | 2019 | BS701_19 | f | ‒ | ‒ | ‒ | ‒ | 0 | -13.18 | 6.82 | -16.97 | 15.96 | -16.01 | 8.36 | -16.22 | 13.66 | 4 |
| Fadila | 2017 | Z440_17 | f | 12.43 | -13.85 | ‒ | ‒ | 1 | -16.65 | 21.42 | -14.54 | 28.83 | ‒ | ‒ | ‒ | ‒ | 2 |
| Fadila | 2018 | BL150_18 | f | -1.68 | 0.14 | 13.61 | -17.43 | 2 | x | x | x | x | x | x | x | x | x |
| Fadila | 2019 | BS727_19 | f | -0.59 | 5.71 | ‒ | ‒ | 1 | -16.46 | 28.52 | -5.36 | 46.75 | ‒ | ‒ | ‒ | ‒ | 2 |
| Finnegan | 2016 | Z451_16 | m | -15.53 | 19.52 | ‒ | ‒ | 1 | -15.10 | 28.03 | ‒ | ‒ | ‒ | ‒ | ‒ | ‒ | 1 |
| Finnegan | 2017 | BD676_17 | m | -15.84 | 19.51 | ‒ | ‒ | 1 | -16.80 | 18.63 | ‒ | ‒ | ‒ | ‒ | ‒ | ‒ | 1 |
| Finnegan | 2018 | BL146_18 | m | -16.41 | 14.39 | ‒ | ‒ | 1 | -16.82 | 17.49 | ‒ | ‒ | ‒ | ‒ | ‒ | ‒ | 1 |
| Finnegan | 2019 | BS722_19 | m | -16.80 | 26.01 | ‒ | ‒ | 1 | -17.14 | 20.79 | -11.93 | 33.17 | ‒ | ‒ | ‒ | ‒ | 2 |
| Fuxia | 2019 | BT686_19 | f | -16.35 | 22.34 | 3.35 | 17.85 | 2 | -17.25 | 20.77 | -7.01 | 45.90 | ‒ | ‒ | ‒ | ‒ | 2 |
| Guinea | 2017 | Z449_17 | f | ‒ | ‒ | ‒ | ‒ | 0 | ‒ | ‒ | ‒ | ‒ | ‒ | ‒ | ‒ | ‒ | 0 |
| Guinea | 2018 | BL149_18 | f | ‒ | ‒ | ‒ | ‒ | 0 | x | x | x | x | x | x | x | x | x |
| Guinea | 2019 | BS716_19 | f | ‒ | ‒ | ‒ | ‒ | 0 | -5.40 | 46.79 | ‒ | ‒ | ‒ | ‒ | ‒ | ‒ | 1 |
| Hanno | 2017 | Z453_17 | m | ‒ | ‒ | ‒ | ‒ | 0 | -10.47 | 36.64 | ‒ | ‒ | ‒ | ‒ | ‒ | ‒ | 1 |
| Hanno | 2018 | BD277_18 | m | ‒ | ‒ | ‒ | ‒ | 0 | -10.52 | 39.01 | ‒ | ‒ | ‒ | ‒ | ‒ | ‒ | 1 |
| Hanno | 2019 | BS717_19 | m | ‒ | ‒ | ‒ | ‒ | 0 | ‒ | ‒ | ‒ | ‒ | ‒ | ‒ | ‒ | ‒ | 0 |
| Hirundo | 2018 | BL152_18 | m | ‒ | ‒ | ‒ | ‒ | 0 | -10.62 | 31.63 | ‒ | ‒ | ‒ | ‒ | ‒ | ‒ | 1 |
| Hirundo | 2019 | BL126_19 | f | ‒ | ‒ | ‒ | ‒ | 0 | -9.66 | 37.99 | -4.71 | 43.95 | ‒ | ‒ | ‒ | ‒ | 2 |
| Humboldt | 2019 | BT688_19 | m | ‒ | ‒ | ‒ | ‒ | 0 | -16.96 | 21.64 | ‒ | ‒ | ‒ | ‒ | ‒ | ‒ | 1 |
| Indira | 2016 | Z438_16 | f | -17.02 | 18.73 | ‒ | ‒ | 1 | -16.12 | 19.11 | -10.12 | 37.19 | ‒ | ‒ | ‒ | ‒ | 2 |
| Indira | 2017 | BD671_17 | f | -17.03 | 17.64 | ‒ | ‒ | 1 | -15.09 | 14.33 | -0.58 | 43.79 | ‒ | ‒ | ‒ | ‒ | 2 |
| Indira | 2018 | BL135_18 | f | -16.18 | 12.63 | ‒ | ‒ | 1 | -16.03 | 11.49 | ‒ | ‒ | ‒ | ‒ | ‒ | ‒ | 1 |
| Indira | 2019 | BS711_19 | f | -16.91 | 23.48 | ‒ | ‒ | 1 | -15.50 | 15.27 | -11.25 | 40.23 | -4.35 | 46.58 | ‒ | ‒ | 3 |
| Jantje | 2016 | Z596_16 | f | -16.86 | 15.51 | 0.56 | 14.75 | 2 | -16.87 | 22.82 | -11.48 | 36.06 | ‒ | ‒ | ‒ | ‒ | 2 |
| Jantje | 2019 | BS713_19 | f | -16.40 | 29.60 | -0.32 | 3.53 | 2 | -17.02 | 20.79 | ‒ | ‒ | ‒ | ‒ | ‒ | ‒ | 1 |
| Joachim | 2017 | Z447_17 | m | ‒ | ‒ | ‒ | ‒ | 0 | -14.86 | 24.47 | ‒ | ‒ | ‒ | ‒ | ‒ | ‒ | 1 |
| Joachim | 2018 | BL129_18 | m | ‒ | ‒ | ‒ | ‒ | 0 | x | x | x | x | x | x | x | x | x |
| Joanne | 2019 | BT687_19 | f | ‒ | ‒ | ‒ | ‒ | 0 | -16.41 | 21.30 | ‒ | ‒ | ‒ | ‒ | ‒ | ‒ | 1 |
| Kirk | 2018 | BL134_18 | m | ‒ | ‒ | ‒ | ‒ | 0 | x | x | x | x | x | x | x | x | x |
| Kirk | 2019 | BS707_19 | m | ‒ | ‒ | ‒ | ‒ | 0 | -17.17 | 20.03 | -6.91 | 45.32 | ‒ | ‒ | ‒ | ‒ | 2 |
| Lamar | 2016 | Z595_16 | m | ‒ | ‒ | ‒ | ‒ | 0 | ‒ | ‒ | ‒ | ‒ | ‒ | ‒ | ‒ | ‒ | 0 |
| Lamar | 2017 | BD678_17 | m | ‒ | ‒ | ‒ | ‒ | 0 | -11.01 | 35.95 | ‒ | ‒ | ‒ | ‒ | ‒ | ‒ | 1 |
| Lamar | 2018 | BL157_18 | m | ‒ | ‒ | ‒ | ‒ | 0 | x | x | x | x | x | x | x | x | x |
| Larkin | 2016 | Z440_16 | m | ‒ | ‒ | ‒ | ‒ | 0 | -9.45 | 42.97 | ‒ | ‒ | ‒ | ‒ | ‒ | ‒ | 1 |
| Larkin | 2017 | BD278_17 | m | ‒ | ‒ | ‒ | ‒ | 0 | x | x | x | x | x | x | x | x | x |
| Larkin | 2018 | BL139_18 | m | ‒ | ‒ | ‒ | ‒ | 0 | x | x | x | x | x | x | x | x | x |
| Laurita | 2019 | BT694_19 | f | -16.84 | 21.46 | ‒ | ‒ | 1 | -15.21 | 17.59 | -18.43 | 27.05 | -9.17 | 47.22 | ‒ | ‒ | 3 |
| Lineka | 2019 | BS726_19 | f | ‒ | ‒ | ‒ | ‒ | 0 | -17.75 | 24.68 | ‒ | ‒ | ‒ | ‒ | ‒ | ‒ | 1 |
| London | 2018 | BD665_18 | m | ‒ | ‒ | ‒ | ‒ | 0 | -11.03 | 31.33 | ‒ | ‒ | ‒ | ‒ | ‒ | ‒ | 1 |
| London | 2019 | BS729_19 | m | ‒ | ‒ | ‒ | ‒ | 0 | ‒ | ‒ | ‒ | ‒ | ‒ | ‒ | ‒ | ‒ | 0 |
| Luca | 2019 | BS737_19 | m | -0.83 | 49.14 | ‒ | ‒ | 1 | -17.41 | 22.66 | ‒ | ‒ | ‒ | ‒ | ‒ | ‒ | 1 |
| Lucius | 2018 | BL164_18 | m | 2.13 | 47.51 | ‒ | ‒ | 1 | -13.76 | 28.03 | ‒ | ‒ | ‒ | ‒ | ‒ | ‒ | 1 |
| Lulu | 2017 | Z436_17 | f | ‒ | ‒ | ‒ | ‒ | 0 | ‒ | ‒ | ‒ | ‒ | ‒ | ‒ | ‒ | ‒ | 0 |
| Lulu | 2018 | BL126_18 | f | ‒ | ‒ | ‒ | ‒ | 0 | ‒ | ‒ | ‒ | ‒ | ‒ | ‒ | ‒ | ‒ | 0 |
| Maigold | 2019 | BS725_19 | f | 4.49 | 51.01 | ‒ | ‒ | 1 | 2.71 | 11.78 | -18.03 | 16.72 | ‒ | ‒ | ‒ | ‒ | 2 |
| Martha | 2018 | BL162_18 | f | ‒ | ‒ | ‒ | ‒ | 0 | 4.82 | 51.37 | ‒ | ‒ | ‒ | ‒ | ‒ | ‒ | 1 |
| Melek | 2017 | Z451_17 | f | -6.79 | 38.79 | -16.79 | 16.79 | 2 | -16.89 | 15.48 | -11.00 | 33.76 | ‒ | ‒ | ‒ | ‒ | 2 |
| Melek | 2018 | BD670_18 | f | -17.28 | 12.85 | ‒ | ‒ | 1 | -14.80 | 25.03 | ‒ | ‒ | ‒ | ‒ | ‒ | ‒ | 1 |
| Merula | 2019 | BT691_19 | f | -17.28 | 22.76 | ‒ | ‒ | 1 | -11.54 | 31.84 | ‒ | ‒ | ‒ | ‒ | ‒ | ‒ | 1 |
| Miranda | 2016 | Z453_16 | f | -7.46 | 35.07 | ‒ | ‒ | 1 | ‒ | ‒ | ‒ | ‒ | ‒ | ‒ | ‒ | ‒ | 0 |
| Miranda | 2017 | BD667_17 | f | -0.93 | 46.54 | ‒ | ‒ | 1 | ‒ | ‒ | ‒ | ‒ | ‒ | ‒ | ‒ | ‒ | 0 |
| Miranda | 2018 | BL147_18 | f | -6.84 | 35.58 | ‒ | ‒ | 1 | x | x | x | x | x | x | x | x | x |
| Miranda | 2019 | BT689_19 | f | -7.97 | 36.92 | ‒ | ‒ | 1 | -6.50 | 45.14 | ‒ | ‒ | ‒ | ‒ | ‒ | ‒ | 1 |
| Moni | 2016 | Z445_16 | f | ‒ | ‒ | ‒ | ‒ | 0 | -8.90 | 44.49 | ‒ | ‒ | ‒ | ‒ | ‒ | ‒ | 1 |
| Moni | 2017 | BD277_17 | f | ‒ | ‒ | ‒ | ‒ | 0 | -10.46 | 40.84 | ‒ | ‒ | ‒ | ‒ | ‒ | ‒ | 1 |
| Moni | 2018 | BL143_18 | f | ‒ | ‒ | ‒ | ‒ | 0 | x | x | x | x | x | x | x | x | x |
| Moni | 2019 | BS721_19 | f | ‒ | ‒ | ‒ | ‒ | 0 | ‒ | ‒ | ‒ | ‒ | ‒ | ‒ | ‒ | ‒ | 0 |
| Nikolas | 2018 | BL154_18 | m | -16.53 | 19.04 | ‒ | ‒ | 1 | x | x | x | x | x | x | x | x | x |
| Nikolas | 2019 | BU573_19 | m | -4.72 | 36.88 | -16.46 | 16.92 | 2 | ‒ | ‒ | ‒ | ‒ | ‒ | ‒ | ‒ | ‒ | 0 |
| Nirmala | 2019 | BS739_19 | f | ‒ | ‒ | ‒ | ‒ | 0 | ‒ | ‒ | ‒ | ‒ | ‒ | ‒ | ‒ | ‒ | 0 |
| Padme | 2018 | BD678_18 | f | -16.75 | 22.05 | ‒ | ‒ | 1 | -17.09 | 17.95 | -10.46 | 32.41 | ‒ | ‒ | ‒ | ‒ | 2 |
| Padme | 2019 | BU571_19 | f | -16.25 | 31.09 | ‒ | ‒ | 1 | x | x | x | x | x | x | x | x | x |
| Pinelopi | 2016 | Z446_16 | f | ‒ | ‒ | ‒ | ‒ | 0 | ‒ | ‒ | ‒ | ‒ | ‒ | ‒ | ‒ | ‒ | 0 |
| Pinelopi | 2017 | BD669_17 | f | ‒ | ‒ | ‒ | ‒ | 0 | -0.45 | 49.21 | ‒ | ‒ | ‒ | ‒ | ‒ | ‒ | 1 |
| Pinelopi | 2018 | BL131_18 | f | ‒ | ‒ | ‒ | ‒ | 0 | x | x | x | x | x | x | x | x | x |
| Pinelopi | 2019 | BS709_19 | f | ‒ | ‒ | ‒ | ‒ | 0 | -17.19 | 12.28 | -15.87 | 26.74 | -11.28 | 40.17 | ‒ | ‒ | 3 |
| Pontus | 2016 | Z597_16 | m | -16.69 | 16.49 | ‒ | ‒ | 1 | x | x | x | x | x | x | x | x | x |
| Pontus | 2017 | Z452_17 | m | ‒ | ‒ | ‒ | ‒ | 0 | x | x | x | x | x | x | x | x | x |
| Pontus | 2018 | BL163_18 | m | ‒ | ‒ | ‒ | ‒ | 0 | x | x | x | x | x | x | x | x | x |
| Pontus | 2019 | BS718_19 | m | ‒ | ‒ | ‒ | ‒ | 0 | -13.97 | 9.48 | -16.50 | 23.25 | ‒ | ‒ | ‒ | ‒ | 2 |
| Primrose | 2019 | BS734_19 | f | ‒ | ‒ | ‒ | ‒ | 0 | x | x | x | x | x | x | x | x | x |
| Princess | 2018 | BD672_18 | f | ‒ | ‒ | ‒ | ‒ | 0 | -11.23 | 37.18 | ‒ | ‒ | ‒ | ‒ | ‒ | ‒ | 1 |
| Princess | 2019 | BS703_19 | f | ‒ | ‒ | ‒ | ‒ | 0 | ‒ | ‒ | ‒ | ‒ | ‒ | ‒ | ‒ | ‒ | 0 |
| Prosecco | 2019 | YVH215_19 | f | ‒ | ‒ | ‒ | ‒ | 0 | -12.50 | 30.86 | ‒ | ‒ | ‒ | ‒ | ‒ | ‒ | 1 |
| Puck | 2018 | BL137_18 | m | 6.86 | 44.30 | -17.08 | 21.91 | 2 | x | x | x | x | x | x | x | x | x |
| Rian | 2016 | Z434_16 | m | ‒ | ‒ | ‒ | ‒ | 0 | ‒ | ‒ | ‒ | ‒ | ‒ | ‒ | ‒ | ‒ | 0 |
| Rian | 2017 | BD679_17 | m | ‒ | ‒ | ‒ | ‒ | 0 | -14.31 | 27.60 | ‒ | ‒ | ‒ | ‒ | ‒ | ‒ | 1 |
| Rian | 2018 | BL144_18 | m | ‒ | ‒ | ‒ | ‒ | 0 | -12.29 | 34.69 | 4.06 | 50.66 | ‒ | ‒ | ‒ | ‒ | 2 |
| Rian | 2019 | BS140_19 | m | ‒ | ‒ | ‒ | ‒ | 0 | -12.06 | 40.74 | ‒ | ‒ | ‒ | ‒ | ‒ | ‒ | 1 |
| Ribanna | 2019 | BS736_19 | f | ‒ | ‒ | ‒ | ‒ | 0 | ‒ | ‒ | ‒ | ‒ | ‒ | ‒ | ‒ | ‒ | 0 |
| Roy | 2016 | Z437_16 | m | ‒ | ‒ | ‒ | ‒ | 0 | x | x | x | x | x | x | x | x | x |
| Roy | 2017 | BD665_17 | m | ‒ | ‒ | ‒ | ‒ | 0 | -16.30 | 19.10 | -14.09 | 30.44 | ‒ | ‒ | ‒ | ‒ | 2 |
| Roy | 2018 | BL128_18 | m | ‒ | ‒ | ‒ | ‒ | 0 | x | x | x | x | x | x | x | x | x |
| Roy | 2019 | BS705_19 | m | ‒ | ‒ | ‒ | ‒ | 0 | -5.13 | 49.18 | ‒ | ‒ | ‒ | ‒ | ‒ | ‒ | 1 |
| Russell | 2019 | BS730_19 | m | ‒ | ‒ | ‒ | ‒ | 0 | -10.48 | 38.30 | ‒ | ‒ | ‒ | ‒ | ‒ | ‒ | 1 |
| Sarina | 2018 | BD667_18 | f | ‒ | ‒ | ‒ | ‒ | 0 | -13.84 | 26.42 | ‒ | ‒ | ‒ | ‒ | ‒ | ‒ | 1 |
| Sarina | 2019 | BS710_19 | f | ‒ | ‒ | ‒ | ‒ | 0 | -17.78 | 26.84 | ‒ | ‒ | ‒ | ‒ | ‒ | ‒ | 1 |
| Satan | 2017 | Z435_17 | m | ‒ | ‒ | ‒ | ‒ | 0 | ‒ | ‒ | ‒ | ‒ | ‒ | ‒ | ‒ | ‒ | 0 |
| Satan | 2018 | BL138_18 | m | ‒ | ‒ | ‒ | ‒ | 0 | x | x | x | x | x | x | x | x | x |
| Satan | 2019 | BS740_19 | m | ‒ | ‒ | ‒ | ‒ | 0 | ‒ | ‒ | ‒ | ‒ | ‒ | ‒ | ‒ | ‒ | 0 |
| Selima | 2019 | BS720_19 | f | ‒ | ‒ | ‒ | ‒ | 0 | ‒ | ‒ | ‒ | ‒ | ‒ | ‒ | ‒ | ‒ | 0 |
| SirDavid | 2016 | Z435_16 | m | ‒ | ‒ | ‒ | ‒ | 0 | ‒ | ‒ | ‒ | ‒ | ‒ | ‒ | ‒ | ‒ | 0 |
| Yealle | 2018 | BL125_18 | f | ‒ | ‒ | ‒ | ‒ | 0 | x | x | x | x | x | x | x | x | x |
| Yealle | 2019 | BS704_19 | f | ‒ | ‒ | ‒ | ‒ | 0 | -15.09 | 31.21 | -11.13 | 44.00 | ‒ | ‒ | ‒ | ‒ | 2 |

**Table S7** Estimated mean longitude and latitude of wintering area(s) and median departure and arrival dates provided by the “*stationary.migration.summary*” function for each common tern deployed with a light-level geolocator in 2016, 2017, 2018 and/or 2019. An “x” indicates the absence of data due to geolocator failure.

| **individual** | **sex** | **year** | **geo** | **departure**  **colony** | **arrival wintering area_1** | **longitude wintering area_1** | **latitude wintering area_1** | **departure wintering area_1** | **arrival wintering areas_2** | **longitude wintering area_2** | **Latitude wintering area_2** | **departure wintering area_2** | **arrival colony** |
| --- | --- | --- | --- | --- | --- | --- | --- | --- | --- | --- | --- | --- | --- |
| Abel | m | 2016 | Z444_16 | 29.08.2016 | 05.09.2016 | -16.77 | 19.87 | 01.04.2017 | – | – | – | – | 09.04.2017 |
| Abel | m | 2017 | BD666_17 | 15.09.2017 | 21.09.2017 | -16.79 | 19.95 | 29.03.2018 | – | – | – | – | 03.04.2018 |
| Abel | m | 2018 | BL127_18 | 04.09.2018 | 08.09.2018 | -16.70 | 20.31 | x | – | – | – | – | x |
| Adelie | f | 2019 | BT693_19 | 26.08.2019 | 31.08.2019 | -16.63 | 18.87 | 07.02.2020 | – | – | – | – | 22.04.2020 |
| Anita | f | 2016 | Z450_16 | 15.09.2016 | 20.09.2016 | -16.66 | 22.32 | 07.04.2017 | – | – | – | – | 11.04.2017 |
| Anita | f | 2017 | BD670_17 | 23.09.2017 | 03.10.2017 | -16.85 | 20.54 | 15.04.2018 | – | – | – | – | 22.04.2018 |
| Anita | f | 2018 | BL161_18 | 07.10.2018 | 16.10.2018 | -16.77 | 20.03 | x | – | – | – | – | x |
| Antoine | m | 2018 | BD668_18 | 13.09.2018 | 20.09.2018 | -16.57 | 15.74 | 08.04.2019 | – | – | – | – | 20.04.2019 |
| Antoine | m | 2019 | BS731_19 | 09.09.2019 | 18.09.2019 | -16.31 | 14.06 | 10.02.2020 | – | – | – | – | 23.04.2020 |
| Aristide | m | 2018 | BL155_18 | 29.08.2018 | 05.09.2018 | -17.21 | 20.70 | x | – | – | – | – | x |
| Armando | m | 2016 | Z447_16 | 26.08.2016 | 02.09.2016 | -17.10 | 15.52 | 28.03.2017 | – | – | – | – | 09.04.2017 |
| Armando | m | 2017 | BD276_17 | 31.08.2017 | 05.09.2017 | -17.19 | 14.51 | 29.03.2018 | – | – | – | – | 13.04.2018 |
| Armando | m | 2018 | BL156_18 | 13.09.2018 | 20.09.2018 | -16.86 | 17.31 | x | – | – | – | – | x |
| Armando | m | 2019 | BS733_19 | 19.09.2019 | 30.09.2019 | -17.08 | 14.26 | 14.04.2020 | – | – | – | – | 23.04.2020 |
| Arno | m | 2017 | Z434_17 | 23.09.2017 | 01.10.2017 | -16.91 | 21.75 | 08.02.2018 | 09.02.2018 | -14.29 | 26.77 | 16.04.2018 | 20.04.2018 |
| Auguste | f | 2016 | Z441_16 | 25.09.2016 | 08.10.2016 | -12.83 | 8.49 | x | – | – | – | – | x |
| Autumn | f | 2019 | BS738_19 | 07.09.2019 | 20.09.2019 | -0.46 | 4.41 | 26.02.2020 | – | – | – | – | 18.04.2020 |
| Bea | f | 2016 | Z452_16 | 31.07.2016 | 06.08.2016 | -17.33 | 21.96 | 28.03.2017 | – | – | – | – | 18.04.2017 |
| Bea | f | 2017 | BD675_17 | 08.08.2017 | 12.08.2017 | -17.03 | 22.05 | x | – | – | – | – | x |
| Beccy | f | 2018 | BL141_18 | 23.09.2018 | 29.09.2018 | -16.16 | 18.92 | 02.04.2019 | – | – | – | – | 11.04.2019 |
| Beccy | f | 2019 | BS702_19 | 21.09.2019 | 03.10.2019 | -16.26 | 17.10 | 04.04.2020 | – | – | – | – | 12.04.2020 |
| Benita | f | 2019 | BT690_19 | 21.09.2019 | 05.10.2019 | -16.93 | 17.18 | 06.04.2020 | – | – | – | – | 22.04.2020 |
| Bibo | m | 2016 | Z594_16 | 30.08.2016 | 07.09.2016 | -16.51 | 20.06 | 04.04.2017 | – | – | – | – | 15.04.2017 |
| Bibo | m | 2017 | BD672_17 | 04.09.2017 | 21.09.2017 | -16.71 | 19.18 | 15.04.2018 | – | – | – | – | 21.04.2018 |
| Bibo | m | 2018 | BL132_18 | 23.09.2018 | 03.10.2018 | -16.44 | 19.23 | x | – | – | – | – | x |
| Bibo | m | 2019 | BS715_19 | 22.09.2019 | 06.10.2019 | -16.58 | 18.98 | 15.04.2020 | – | – | – | – | 23.04.2020 |
| Blake | m | 2017 | Z446_17 | 23.08.2017 | 02.09.2017 | -16.85 | 18.58 | 14.04.2018 | – | – | – | – | 19.04.2018 |
| Blake | m | 2018 | BL145_18 | 24.09.2018 | 28.09.2018 | -16.38 | 19.40 | x | – | – | – | – | x |
| Blake | m | 2019 | BS714_19 | 13.08.2019 | 30.08.2019 | -16.54 | 19.13 | 05.04.2020 | – | – | – | – | 16.04.2020 |
| Bofur | m | 2019 | BS724_19 | 11.09.2019 | 19.09.2019 | -16.33 | 11.88 | 02.04.2020 | – | – | – | – | 20.04.2020 |
| Cosima | f | 2016 | Z443_16 | 25.07.2016 | 09.08.2016 | 0.82 | 5.46 | 11.03.2017 | – | – | – | – | 01.04.2017 |
| Cosima | f | 2017 | BD680_17 | 01.08.2017 | 19.08.2017 | 0.77 | 6.38 | 16.03.2018 | – | – | – | – | 07.04.2018 |
| Cosima | f | 2018 | BL158_18 | 05.08.2018 | 19.08.2018 | 0.90 | 5.34 | x | – | – | – | – | x |
| Cosima | f | 2019 | BS708_19 | 09.08.2019 | 28.08.2019 | 1.03 | 5.36 | x | – | – | – | – | x |
| Cupido | m | 2019 | BS735_19 | 25.08.2019 | 21.09.2019 | 13.97 | -23.05 | 26.03.2020 | – | – | – | – | 04.05.2020 |
| Danilo | m | 2016 | Z449_16 | 12.09.2016 | 28.09.2016 | 0.00 | 5.57 | 28.03.2017 | – | – | – | – | 15.04.2017 |
| Danilo | m | 2017 | BD668_17 | 22.09.2017 | 14.10.2017 | -0.24 | 5.41 | 31.03.2018 | – | – | – | – | 20.04.2018 |
| Danilo | m | 2018 | BL153_18 | 24.09.2018 | 05.10.2018 | -0.45 | 4.98 | x | – | – | – | – | x |
| Djamila | f | 2016 | Z448_16 | 24.07.2016 | 01.08.2016 | -17.00 | 20.48 | 03.12.2016 | 07.12.2016 | -14.12 | 10.86 | 21.03.2017 | 15.04.2017 |
| Djamila | f | 2017 | BD673_17 | 22.08.2017 | 19.10.2017 | -13.78 | 9.27 | 06.04.2018 | – | – | – | – | 01.05.2018 |
| Djamila | f | 2018 | BD679_18 | 30.08.2018 | 04.09.2018 | -16.86 | 21.27 | 06.11.2018 | 06.11.2018 | -13.93 | 9.61 | 01.04.2019 | 23.04.2019 |
| Djamila | f | 2019 | BS728_19 | 23.08.2019 | 29.10.2019 | -13.81 | 9.68 | 11.04.2020 | – | – | – | – | 29.04.2020 |
| Elvira | f | 2016 | Z436_16 | 27.08.2016 | 08.09.2016 | -15.99 | 11.39 | 10.02.2017 | – | – | – | – | 15.04.2017 |
| Elvira | f | 2017 | BD664_17 | 28.08.2017 | 06.09.2017 | -15.53 | 10.36 | 20.03.2018 | – | – | – | – | 18.04.2018 |
| Elvira | f | 2018 | BL133_18 | 30.08.2018 | 06.09.2018 | -15.55 | 10.76 | 21.02.2019 | – | – | – | – | x |
| Elvira | f | 2019 | BS701_19 | 08.09.2019 | 20.09.2019 | -16.04 | 10.72 | 14.01.2020 | – | – | – | – | 22.04.2020 |
| Fadila | f | 2017 | Z440_17 | 05.08.2017 | 15.09.2017 | 18.61 | -35.27 | 21.03.2018 | – | – | – | – | 16.04.2018 |
| Fadila | f | 2018 | BL150_18 | 29.08.2018 | 13.10.2018 | 17.72 | -33.60 | x | – | – | – | – | x |
| Fadila | f | 2019 | BS727_19 | 26.08.2019 | 30.09.2019 | 18.41 | -34.05 | 20.03.2020 | – | – | – | – | 19.04.2020 |
| Finnegan | m | 2016 | Z451_16 | 12.09.2016 | 30.09.2016 | 0.26 | 7.27 | 27.03.2017 | – | – | – | – | 18.04.2017 |
| Finnegan | m | 2017 | BD676_17 | 23.09.2017 | 13.10.2017 | 0.41 | 5.54 | 23.03.2018 | – | – | – | – | 01.05.2018 |
| Finnegan | m | 2018 | BL146_18 | 05.10.2018 | 28.10.2018 | 0.11 | 5.93 | 22.03.2019 | – | – | – | – | 24.04.2019 |
| Finnegan | m | 2019 | BS722_19 | 01.10.2019 | 16.10.2019 | 0.44 | 4.64 | 27.03.2020 | – | – | – | – | 19.04.2020 |
| Fuxia | f | 2019 | BT686_19 | 08.09.2019 | 03.10.2019 | 6.45 | 5.44 | 22.03.2020 | – | – | – | – | 24.04.2020 |
| Guinea | f | 2017 | Z449_17 | 24.07.2017 | 26.07.2017 | -16.67 | 18.22 | 30.03.2018 | – | – | – | – | 08.04.2018 |
| Guinea | f | 2018 | BL149_18 | 30.08.2018 | 04.09.2018 | -16.35 | 19.03 | x | – | – | – | – | x |
| Guinea | f | 2019 | BS716_19 | 21.08.2019 | 26.08.2019 | -16.64 | 17.84 | 30.03.2020 | – | – | – | – | 17.04.2020 |
| Hanno | m | 2017 | Z453_17 | 31.08.2017 | 07.09.2017 | -17.19 | 14.49 | 17.04.2018 | – | – | – | – | 01.05.2018 |
| Hanno | m | 2018 | BD277_18 | 04.09.2018 | 09.09.2018 | -17.00 | 14.60 | 14.04.2019 | – | – | – | – | 24.04.2019 |
| Hanno | m | 2019 | BS717_19 | 06.09.2019 | 11.09.2019 | -17.08 | 12.94 | 12.04.2020 | – | – | – | – | 29.04.2020 |
| Hirundo | m | 2018 | BL152_18 | 24.09.2018 | 03.10.2018 | -16.50 | 17.54 | 02.04.2019 | – | – | – | – | 18.04.2019 |
| Hirundo | f | 2019 | BL126_19 | 01.10.2019 | 09.10.2019 | -16.65 | 19.16 | 04.04.2020 | – | – | – | – | 20.04.2020 |
| Humboldt | m | 2019 | BT688_19 | 27.09.2019 | 05.10.2019 | -16.58 | 12.24 | 19.03.2020 | – | – | – | – | 01.05.2020 |
| Indira | f | 2016 | Z438_16 | 23.08.2016 | 08.09.2016 | 0.05 | 6.19 | 17.03.2017 | – | – | – | – | 13.04.2017 |
| Indira | f | 2017 | BD671_17 | 30.08.2017 | 17.09.2017 | -0.23 | 6.06 | 01.03.2018 | – | – | – | – | 15.04.2018 |
| Indira | f | 2018 | BL135_18 | 12.09.2018 | 28.09.2018 | 0.01 | 5.40 | 26.03.2019 | – | – | – | – | 22.04.2019 |
| Indira | f | 2019 | BS711_19 | 09.09.2019 | 25.09.2019 | 0.11 | 5.27 | 18.03.2020 | – | – | – | – | 23.04.2020 |
| Jantje | f | 2016 | Z596_16 | 05.09.2016 | 20.10.2016 | 14.02 | -22.82 | 21.03.2017 | – | – | – | – | 12.04.2017 |
| Jantje | f | 2019 | BS713_19 | 17.09.2019 | 22.10.2019 | 14.22 | -24.68 | 16.03.2020 | – | – | – | – | 12.04.2020 |
| Joachim | m | 2017 | Z447_17 | 21.09.2017 | 01.10.2017 | -17.27 | 15.56 | 10.04.2018 | – | – | – | – | 19.04.2018 |
| Joachim | m | 2018 | BL129_18 | 28.09.2018 | 03.10.2018 | -17.31 | 15.27 | x | – | – | – | – | x |
| Joanne | f | 2019 | BT687_19 | 07.09.2019 | 11.09.2019 | -16.26 | 13.30 | 16.04.2020 | – | – | – | – | 30.04.2020 |
| Kirk | m | 2018 | BL134_18 | 24.09.2018 | 29.09.2018 | -16.46 | 22.94 | x | – | – | – | – | x |
| Kirk | m | 2019 | BS707_19 | 17.09.2019 | 26.09.2019 | -16.62 | 17.17 | 13.03.2020 | – | – | – | – | 17.04.2020 |
| Lamar | m | 2016 | Z595_16 | 13.09.2016 | 16.09.2016 | -16.46 | 20.51 | 19.04.2017 | – | – | – | – | 30.04.2017 |
| Lamar | m | 2017 | BD678_17 | 21.09.2017 | 27.09.2017 | -16.86 | 18.39 | 22.04.2018 | – | – | – | – | 04.05.2018 |
| Lamar | m | 2018 | BL157_18 | 24.09.2018 | 28.09.2018 | -16.68 | 19.05 | x | – | – | – | – | x |
| Larkin | m | 2016 | Z440_16 | 26.08.2016 | 02.09.2016 | -16.52 | 14.76 | 06.04.2017 | – | – | – | – | 25.04.2017 |
| Larkin | m | 2017 | BD278_17 | 01.09.2017 | 09.09.2017 | -16.28 | 12.84 | x | – | – | – | – | x |
| Larkin | m | 2018 | BL139_18 | 12.09.2018 | 16.09.2018 | -16.67 | 16.24 | x | – | – | – | – | x |
| Laurita | f | 2019 | BT694_19 | 24.08.2019 | 07.09.2019 | -0.49 | 5.84 | 18.03.2020 | – | – | – | – | 25.04.2020 |
| Lineka | f | 2019 | BS726_19 | 29.08.2019 | 04.09.2019 | -16.76 | 18.78 | 11.03.2020 | – | – | – | – | 07.04.2020 |
| London | m | 2018 | BD665_18 | 01.10.2018 | 11.10.2018 | -16.90 | 17.15 | 14.04.2019 | – | – | – | – | 30.04.2019 |
| London | m | 2019 | BS729_19 | 01.10.2019 | 06.10.2019 | -16.68 | 17.38 | 06.04.2020 | – | – | – | – | 19.04.2020 |
| Luca | m | 2019 | BS737_19 | 21.09.2019 | 21.10.2019 | -16.47 | 10.43 | 03.04.2020 | – | – | – | – | 19.04.2020 |
| Lucius | m | 2018 | BL164_18 | 17.09.2018 | 26.09.2018 | -16.87 | 19.10 | 16.03.2019 | – | – | – | – | 20.04.2019 |
| Lulu | f | 2017 | Z436_17 | 25.08.2017 | 29.08.2017 | -17.60 | 16.51 | 05.04.2018 | – | – | – | – | 13.04.2018 |
| Lulu | f | 2018 | BL126_18 | 19.09.2018 | 26.09.2018 | -17.26 | 17.04 | 08.04.2019 | – | – | – | – | 21.04.2019 |
| Maigold | f | 2019 | BS725_19 | 18.08.2019 | 05.09.2019 | 0.71 | 6.19 | 07.03.2020 | – | – | – | – | 22.04.2020 |
| Martha* | f | 2018 | BL162_18 | 29.08.2018 | 03.09.2018 | -16.79 | 13.97 | x | x | -16.33 | 19.80 | 25.03.2019 | 26.04.2019 |
| Melek | f | 2017 | Z451_17 | 29.08.2017 | 16.09.2017 | -0.65 | 4.55 | 06.04.2018 | – | – | – | – | 16.05.2018 |
| Melek | f | 2018 | BD670_18 | 23.08.2018 | 18.09.2018 | -0.46 | 4.64 | 28.03.2019 | – | – | – | – | 08.05.2019 |
| Merula | f | 2019 | BT691_19 | 23.08.2019 | 04.09.2019 | -15.72 | 10.69 | 25.04.2020 | – | – | – | – | 07.05.2020 |
| Miranda | f | 2016 | Z453_16 | 29.08.2016 | 10.09.2016 | -16.43 | 13.21 | 06.04.2017 | – | – | – | – | 15.04.2017 |
| Miranda | f | 2017 | BD667_17 | 31.08.2017 | 23.09.2017 | -16.62 | 13.24 | 12.04.2018 | – | – | – | – | 22.04.2018 |
| Miranda | f | 2018 | BL147_18 | 02.09.2018 | 13.09.2018 | -16.51 | 12.81 | x | – | – | – | – | x |
| Miranda | f | 2019 | BT689_19 | 01.09.2019 | 20.09.2019 | -16.79 | 14.48 | 30.03.2020 | – | – | – | – | 19.04.2020 |
| Moni | f | 2016 | Z445_16 | 25.08.2016 | 29.08.2016 | -16.77 | 20.02 | 03.04.2017 | – | – | – | – | 16.04.2017 |
| Moni | f | 2017 | BD277_17 | 01.09.2017 | 06.09.2017 | -16.70 | 19.10 | 31.03.2018 | – | – | – | – | 17.04.2018 |
| Moni | f | 2018 | BL143_18 | 30.08.2018 | 03.09.2018 | -16.90 | 20.84 | x | – | – | – | – | x |
| Moni | f | 2019 | BS721_19 | 27.08.2019 | 01.09.2019 | -16.58 | 18.29 | 31.03.2020 | – | – | – | – | 10.04.2020 |
| Nikolas | m | 2018 | BL154_18 | 28.09.2018 | 30.10.2018 | -16.80 | 12.30 | x | – | – | – | – | x |
| Nikolas | m | 2019 | BU573_19 | 30.09.2019 | 15.11.2019 | -16.48 | 10.84 | 15.04.2020 | – | – | – | – | 28.04.2020 |
| Nirmala | f | 2019 | BS739_19 | 05.09.2019 | 09.09.2019 | -16.57 | 19.02 | 09.04.2020 | – | – | – | – | 23.04.2020 |
| Padme | f | 2018 | BD678_18 | 12.09.2018 | 26.09.2018 | -0.30 | 5.20 | 08.04.2019 | – | – | – | – | 07.05.2019 |
| Padme | f | 2019 | BU571_19 | 07.09.2019 | 29.09.2019 | -0.07 | 3.24 | 28.03.2020 | – | – | – | – | x |
| Pinelopi | f | 2016 | Z446_16 | 30.08.2016 | 07.09.2016 | -16.49 | 19.33 | 02.04.2017 | – | – | – | – | 10.04.2017 |
| Pinelopi | f | 2017 | BD669_17 | 01.09.2017 | 07.09.2017 | -16.72 | 18.32 | 31.03.2018 | – | – | – | – | 13.04.2018 |
| Pinelopi | f | 2018 | BL131_18 | 30.08.2018 | 05.09.2018 | -16.73 | 17.83 | x | – | – | – | – | x |
| Pinelopi | f | 2019 | BS709_19 | 25.08.2019 | 30.08.2019 | -16.50 | 17.71 | 26.02.2020 | – | – | – | – | 06.04.2020 |
| Pontus | m | 2016 | Z597_16 | 24.08.2016 | 05.09.2016 | 0.40 | 7.98 | x | – | – | – | – | x |
| Pontus | m | 2017 | Z452_17 | 23.08.2017 | 08.09.2017 | -0.05 | 6.42 | x | – | – | – | – | x |
| Pontus | m | 2018 | BL163_18 | 31.08.2018 | 15.09.2018 | 0.44 | 7.74 | x | – | – | – | – | x |
| Pontus | m | 2019 | BS718_19 | 05.09.2019 | 16.09.2019 | 0.82 | 5.05 | 24.03.2020 | – | – | – | – | 08.05.2020 |
| Primrose | f | 2019 | BS734_19 | 27.08.2019 | 03.09.2019 | -16.63 | 19.46 | x | – | – | – | – | – |
| Princess | f | 2018 | BD672_18 | 30.08.2018 | 04.09.2018 | -16.62 | 17.70 | 27.03.2019 | – | – | – | – | 08.04.2019 |
| Princess | f | 2019 | BS703_19 | 22.08.2019 | 27.08.2019 | -16.50 | 18.03 | 27.03.2020 | – | – | – | – | 06.04.2020 |
| Prosecco | f | 2019 | YVH215_19 | 02.09.2019 | 08.09.2019 | -16.46 | 11.14 | 21.04.2020 | – | – | – | – | 02.05.2020 |
| Puck | m | 2018 | BL137_18 | 15.09.2018 | 03.10.2018 | -0.47 | 6.20 | 27.12.2018 | 28.12.2018 | 5.43 | 8.17 | x | x |
| Rian | m | 2016 | Z434_16 | 11.09.2016 | 15.09.2016 | -16.77 | 22.57 | 30.03.2017 | – | – | – | – | 10.04.2017 |
| Rian | m | 2017 | BD679_17 | 16.09.2017 | 22.09.2017 | -16.95 | 21.27 | 23.03.2018 | – | – | – | – | 05.04.2018 |
| Rian | m | 2018 | BL144_18 | 17.09.2018 | 24.09.2018 | -16.84 | 20.40 | 24.03.2019 | – | – | – | – | 15.04.2019 |
| Rian | m | 2019 | BS140_19 | 21.09.2019 | 03.10.2019 | -17.03 | 19.31 | 24.03.2020 | – | – | – | – | 07.04.2020 |
| Ribanna | f | 2019 | BS736_19 | 07.09.2019 | 11.09.2019 | -17.22 | 15.10 | 31.03.2020 | – | – | – | – | 23.04.2020 |
| Roy | m | 2016 | Z437_16 | 10.09.2016 | 20.09.2016 | -13.42 | 11.01 | x | – | – | – | – | x |
| Roy | m | 2017 | BD665_17 | 18.09.2017 | 29.09.2017 | -13.66 | 8.98 | 30.03.2018 | – | – | – | – | 18.04.2018 |
| Roy | m | 2018 | BL128_18 | 23.09.2018 | 01.10.2018 | -13.32 | 12.82 | x | – | – | – | – | x |
| Roy | m | 2019 | BS705_19 | 18.09.2019 | 30.09.2019 | -13.30 | 8.50 | 31.03.2020 | – | – | – | – | 22.04.2020 |
| Russell | m | 2019 | BS730_19 | 10.09.2019 | 24.09.2019 | -16.87 | 17.70 | 16.03.2020 | – | – | – | – | 11.04.2020 |
| Sarina | f | 2018 | BD667_18 | 06.08.2018 | 17.08.2018 | -16.59 | 15.13 | 09.04.2019 | – | – | – | – | 21.04.2019 |
| Sarina | f | 2019 | BS710_19 | 03.08.2019 | 14.08.2019 | -16.50 | 15.32 | 06.04.2020 | – | – | – | – | 22.04.2020 |
| Satan | m | 2017 | Z435_17 | 31.08.2017 | 07.09.2017 | -16.24 | 12.69 | 10.04.2018 | – | – | – | – | 18.04.2018 |
| Satan | m | 2018 | BL138_18 | 12.09.2018 | 19.09.2018 | -16.60 | 12.68 | x | – | – | – | – | x |
| Satan | m | 2019 | BS740_19 | 25.09.2019 | 09.10.2019 | -16.13 | 11.76 | 15.04.2020 | – | – | – | – | 19.04.2020 |
| Selima | f | 2019 | BS720_19 | 23.08.2019 | 27.08.2019 | -16.94 | 21.59 | 01.04.2020 | – | – | – | – | 06.04.2020 |
| SirDavid | m | 2016 | Z435_16 | 09.09.2016 | 16.09.2016 | -15.10 | 11.73 | 03.04.2017 | – | – | – | – | 11.04.2017 |
| Yealle | f | 2018 | BL125_18 | 21.08.2018 | 31.08.2018 | -16.79 | 15.33 | x | – | – | – | – | x |
| Yealle | f | 2019 | BS704_19 | 25.08.2019 | 30.08.2019 | -16.62 | 13.49 | 13.03.2020 | – | – | – | – | 05.04.2020 |

*Note: due to a temporary geolocator failure in the wintering areas, the “*stationary.migration.summary*” function could not detect the departure date from the first wintering area and the arrival date at the second wintering area.

**
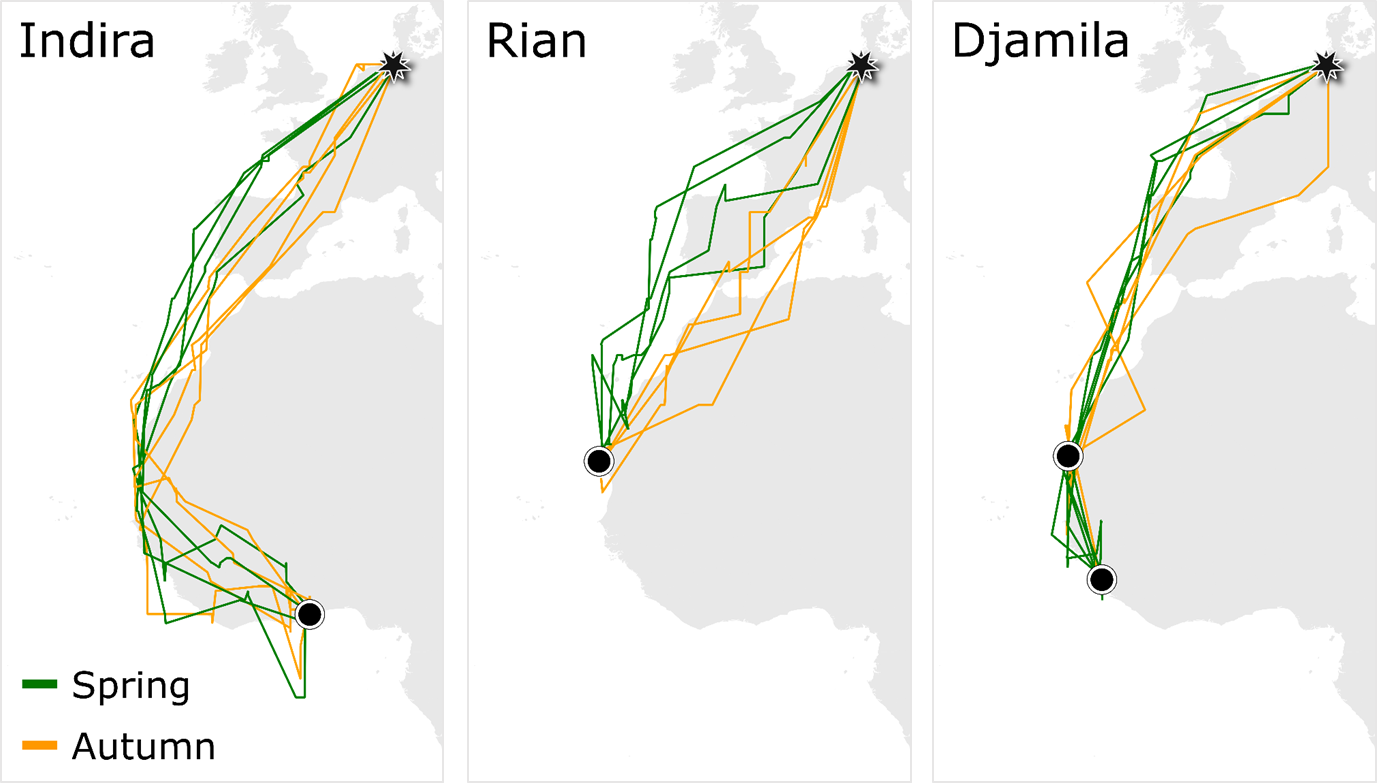
**

**Figure S1** Autumn (orange) and spring (green) migration routes of Indira (female), Rian (male) and Djamila (female) deployed with light-level geolocators in 2016, 2017, 2018, and 2019. The star indicates the breeding colony at the Banter See in Wilhelmshaven (Germany), the dot the wintering area(s) in Africa.
