# Supplementary material for: High individual repeatability of the migratory behaviour of a long-distance migratory seabird
Source: Mov Ecol. 2022 Feb 5;10:5. doi: 10.1186/s40462-022-00303-y (PMC8817581; doi:10.1186/s40462-022-00303-y)
Supplement: Supplementary file 2 — Additional file 2. R code for the analysis of our light-level geolocator data. [file 40462_2022_303_MOESM2_ESM.docx]

Additional file 2

for

**High individual consistency in the migratory behaviour of a long-distance migratory seabird**

by

Nathalie Kürten, Heiko Schmaljohann, Coraline Bichet, Birgen Haest, Oscar Vedder, Jacob González-Solís, Sandra Bouwhuis

R code for the analysis of our light-level geolocator data:

**# 1. Libraries**

library(devtools)

library(FLightR)

library(BAStag)

library(dplyr)

library(maptools)

library(ggmap)

library(grid)

library(shape)

**# 2. General code**

*# set time to GMT*

Sys.setenv(tz="GMT")

*# define and set work directory*

wd <- "my_working_directory"

setwd(wd)

getwd()

*# google key*

api.key <- c("my_API_key")

register_google(key=api.key, account_type = "standard")

**# 3. Bird data**

bird.dat <- data.frame(birdID = c("Z440_16", "Z434_16", "Z436_16",…),

cap_date = c("2016-05-20", "2016-05-18", NA,…),

endinc_date = c("2016-06-01", "2016-05-30", "2016-07-19",…),

lastreg_date = c("2016-08-16", "2016-07-12", "2016-08-23"),

firstreg_date = c("2017-05-13", "2017-04-08", "2017-04-16",…),

startinc_date = c("2017-05-16", "2017-05-27", "2017-05-16",…),

recap_date =c("2017-05-29", "2017-06-07", "2017-05-29"…),

end.first.cal.per = c("2016-08-25", "2016-08-25", "2016-08-28",…),

start.sec.cal.per = c("2017-04-25", "2017-04-04", "2017-04-16",…),

sex = c("m", "m", "f",…),

year = c("2016", "2016", "2016",…),

long_breed = c(8 + 6/60),

lat_breed = c(53+30/60))

bird.dat$cap_date <- as.POSIXct(bird.dat$cap_date, tz = "GMT")

bird.dat$endinc_date <- as.POSIXct(bird.dat$endinc_date, tz = "GMT")

bird.dat$lastreg_date <- as.POSIXct(bird.dat$lastreg_date, tz = "GMT")

bird.dat$firstreg_date <- as.POSIXct(bird.dat$firstreg_date, tz = "GMT")

bird.dat$startinc_date <- as.POSIXct(bird.dat$startinc_date, tz = "GMT")

bird.dat$recap_date <- as.POSIXct(bird.dat$recap_date, tz = "GMT")

bird.dat$end.first.cal.per <- as.POSIXct(bird.dat$end.first.cal.per, tz = "GMT")

bird.dat$start.sec.cal.per <- as.POSIXct(bird.dat$start.sec.cal.per, tz = "GMT")

str(bird.dat)

**# 4. Definition of twilight events**

*# select bird according to the bird id*

id <- bird.dat$birdID[1]

id

*# read the lux data*

d.lux <- readMTlux(paste0(id,".","lux"))

d.lux <- subset(d.lux,select=c("Date","Light"))

str(d.lux)

*# look at the light-level data*

offset = 12

lightImage(d.lux, offset = offset, zlim = c(0, 12), dt = 300)

start <- bird.dat$cap_date[bird.dat$birdID==id]

lastreg <- bird.dat$lastreg_date[bird.dat$birdID==id]

firstreg <- bird.dat$firstreg_date[bird.dat$birdID==id]

end <- bird.dat$recap[bird.dat$birdID==id]

abline(v = c(start, lastreg, firstreg, end), lty = c(1,2,2,1),

col = c("orange", "blue", "blue", "orange"), lwd = 10)

mtext(text=id, side=3, line=1, cex=1.5)

*# restrict data to time until recapture at the breeding area*

d.lux <- subset(d.lux,

Date >= bird.dat$endinc_date[bird.dat$birdID==id] &

Date <= bird.dat$startinc_date[bird.dat$birdID==id])

threshold <- 1.5 *# for Intigeo tags*

twl <- preprocessLight(d.lux, threshold, offset = 12, lmax = 5)

str(twl)

# t*ransfer BAStag output to TAGS format*

TAGS.twilights.raw <- BAStag2TAGS(d.lux, twl, threshold = threshold)

TAGS.twilights.raw$datetime <- format(TAGS.twilights.raw$datetime, format="%Y-%m-%dT%T.000Z")

write.csv(TAGS.twilights.raw, file=paste0(id,".","csv"), quote=FALSE, row.names=FALSE)

TAGS.twilights.raw <- read.csv(file=paste0(id,".","csv"))

**# 5. FLightR - analysis steps**

*# select bird according to the bird id*

id <- bird.dat$birdID[1]

id

Proc.data <- get.tags.data(paste0(id,".","csv"))

*# i. Calibration*

*# find calibration periods for a known calibration location*

par(mfrow=c(1,1), mar=rep(4,4))

plot_slopes_by_location(Proc.data=Proc.data, location=c(bird.dat$long_breed[bird.dat$birdID==id],

bird.dat$lat_breed[bird.dat$birdID==id]))

*# play with abline() to find the proper boundaries for the calibration*

abline(v=as.POSIXct("1999-01-01")) *# end of first calibration period*

abline(v=as.POSIXct("1999-01-01")) *# start of the second calibration period*

*# include end of first and start of the second calibration period*

bird.dat$end.first.cal.per[bird.dat$birdID == id] <- as.POSIXct("1999-01-01", tz = "GMT")

bird.dat$start.sec.cal.per[bird.dat$birdID == id] <- as.POSIXct("1999-01-01", tz = "GMT")

###########################

*# start of the loop*

for (i in c(1, 2, 3,…))

{

#i <- 1 *# to select one bird*

id <- bird.dat$birdID[i]

*# load twilight and raw data*

Proc.data <- get.tags.data(paste0(id,".","csv"))

str(Proc.data)

Calibration.periods <- data.frame(calibration.start = as.POSIXct(c(NA, bird.dat$start.sec.cal.per[bird.dat$birdID
 == id]), origin="1970-01-01"),

calibration.stop = as.POSIXct(c(bird.dat$end.first.cal.per[bird.dat$birdID

== id], NA), origin="1970-01-01"),

lon = bird.dat$long_breed[bird.dat$birdID==id],

lat = bird.dat$lat_breed[bird.dat$birdID==id])

Calibration <- make.calibration(Proc.data, Calibration.periods)

*# ii. Assign spatial extent*

Grid <- make.grid(left = -40, bottom = -45, right = 45, top = 65,

distance.from.land.allowed.to.use = c(-Inf, Inf),

distance.from.land.allowed.to.stay = c(-Inf, Inf))

*# iii. Prepare the model for run*

all.in <- make.prerun.object(Proc.data, Grid, start=c(bird.dat$long_breed[bird.dat$birdID==id],

bird.dat$lat_breed[bird.dat$birdID==id]),

Calibration=Calibration,

M.mean = 50*24,

M.sd = 300)

*# iv. Particle filter run*

nParticles <- 1e^6^

Result <- run.particle.filter(all.in, threads=-1,

nParticles=nParticles,

known.last=TRUE,

precision.sd=25,

check.outliers=F)

save(Result, file = paste0(id,".","RData"))

load(paste0(id,".","RData"))

**# 6. Results**

*# visualisation of the results*

png(filename=paste0("my_working_directory ",id,"_","map",".","png"), width=800, height=800) map.FLightR.ggmap(Result, zoom = 3 ),

dev.off()

}

# end of the loop

###########################
